# Avian influenza amplified age-related mortality in a long-lived seabird

**DOI:** 10.1038/s41467-026-76726-7

**Published:** 2026-08-19

**Authors:** Wouter Courtens, Mónika Ballmann, Ruben Fijn, Morten Frederiksen, Susanne Kühn, Luc Lens, Mardik Leopold, Sander Lilipaly, Florian Packmor, Eric W. M. Stienen, Peter van Horssen, Ulrich Knief

**Affiliations:** 1 Research Institute for Nature and Forest, Brussels, Belgium; 2 Department of Biology, Ghent University, Ghent, Belgium; 3 Wageningen Bioveterinary Research, Lelystad, The Netherlands; 4 Deltamilieu Projecten, Vlissingen, The Netherlands; 5 Department of Bird Ecology, Waardenburg Ecology, Culemborg, The Netherlands; 6 Department of Ecoscience, Aarhus University, Roskilde, Denmark; 7 Wageningen Marine Research, Den Helder, The Netherlands; 8 Lower Saxon Wadden Sea National Park Authority, Wilhelmshaven, Germany; 9 Greenstat, Tricht, The Netherlands; 10 Evolutionary Biology & Ecology, University of Freiburg, Freiburg, Germany

**Keywords:** Population dynamics, Conservation biology

## Abstract

In recent years, highly pathogenic avian influenza (HPAI) A (H5N1) clade 2.3.4.4b viruses have caused mass mortality events in seabirds worldwide, raising concern for long-lived species with low reproductive rates. Using 28 years of ringing data together with individual-level data from the 2022 mass mortality event in northwestern European Sandwich terns (*Thalasseus sandvicensis*), we show that mortality was biased towards older individuals, while no sex bias was observed. HPAI-related mortality increased from approximately 5–10% in young breeders to more than 40% in the oldest individuals. This age-biased loss likely removed the most experienced individuals from the population. Our findings highlight a previously underappreciated mechanism through which HPAI outbreaks may impair the resilience of long-lived avian populations.

## Abstract (web summary)

In this study, the authors show that during the 2022 avian influenza outbreak, Sandwich tern mortality rose sharply with age: over 40% of the oldest birds died versus 5–10% of young breeders, likely removing experienced individuals and potentially weakening population resilience.

## Introduction

Long-lived seabirds are characterized by delayed maturation, low reproductive output, and high adult survival^1^. Because of these life-history characteristics, factors that increase adult mortality have a greater negative impact on population size than reductions in reproductive success or juvenile survival^2–4^. When the highly pathogenic avian influenza (HPAI) A (H5N1) clade 2.3.4.4b virus became enzootic in northwestern Europe in 2021^5^, it caused massive die-offs of adult seabirds^6^. Globally, more than 100 species of seabirds – including several endangered taxa – have been affected by this panzootic^7^, with total mortality in wild birds amounting to at least 1.4 million individuals^8^. While these numbers already raise concerns about the ability of many species to recover^9^, the long-term impacts and prospects for recovery will largely depend on which demographic components are most affected^10^.

Age- or sex-biased mortality can influence population dynamics in long-lived species, but the extent and direction of this effect depend on how survival and reproduction vary across age classes^11^. In birds, older individuals often contribute disproportionately to population growth through greater breeding performance driven by accumulated experience or selective survival of high-quality individuals^12,13^. Additionally, sex-biased mortality can have substantial effects on population dynamics, especially in seabirds with monogamous mating systems and obligate biparental care^14^. Understanding these demographic processes is thus essential for predicting population recovery after mortality events.

In 2022, the Sandwich tern (*Thalasseus sandvicensis*), a seabird with a recorded lifespan of more than 30 years, was one of the bird species severely affected by HPAI in northwestern Europe, losing over 20,500 adults ( > 17% of its regional population^15^). The virus spread rapidly through the densely populated colonies, where intense contact between breeding as well as prospecting birds likely facilitated transmission, both within and between colonies^16^. Here, we show that the 2022 mass mortality event was age- but not sex-biased in Sandwich terns.

## Results

### Sex-specific mortality

Sex-specific mortality was assessed by sexing 243 dead adult Sandwich terns of known age, collected in the Netherlands during the 2022 outbreak. We observed an almost equal sex ratio (123 females, 120 males), with no significant deviation from 1:1 (binomial test, *P* = 0.90), and found no evidence that the age distribution differed between males and females (Wilcoxon rank-sum test, *W* = 4412.5, *P* = 0.33).

### Age-related mortality

**Fig. 1:**
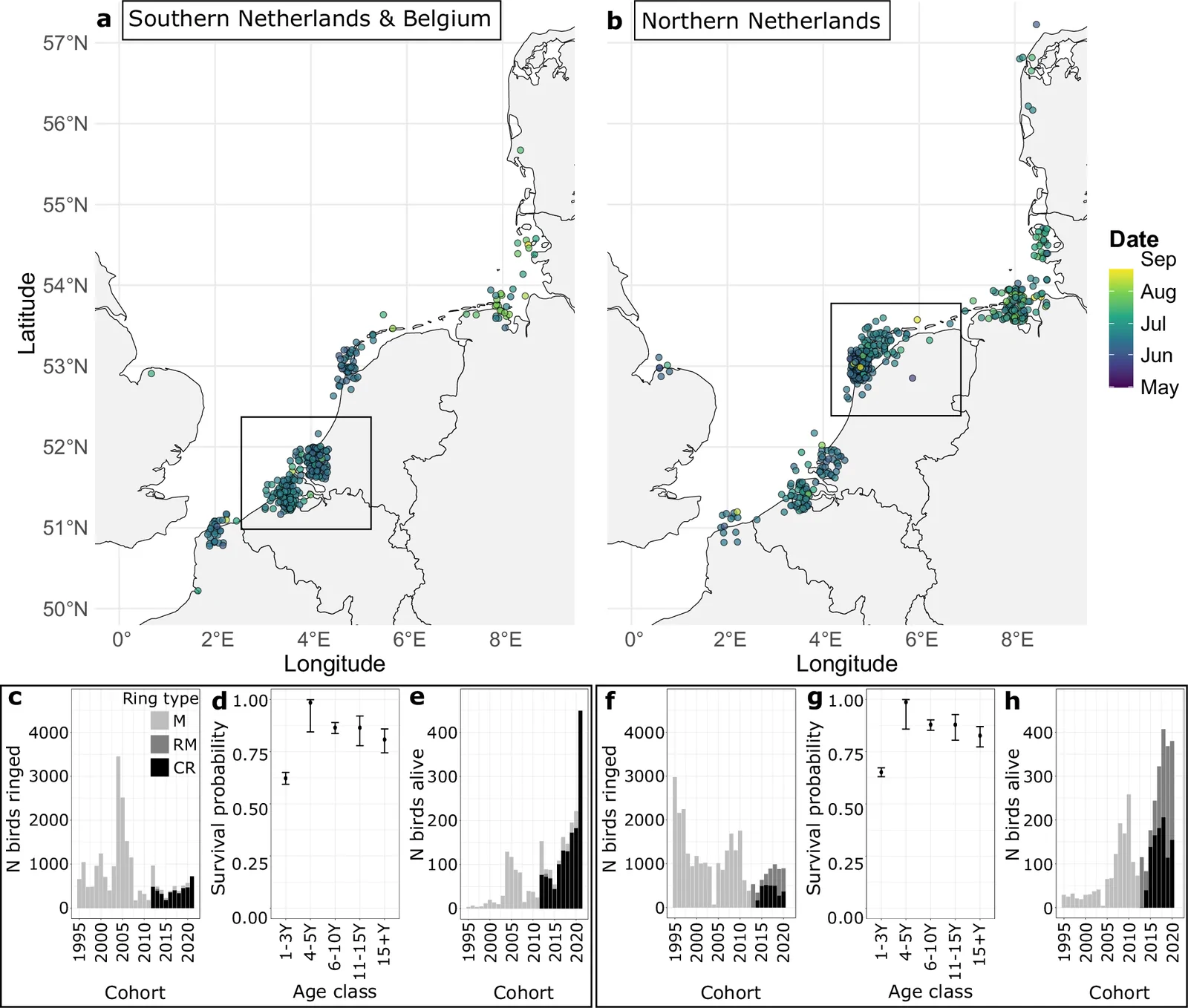
Distribution and demographic context of HPAI-related mortality in ringed Sandwich terns. Spatial distribution of dead Dutch/Belgian ringed Sandwich terns following HPAI A (H5N1) virus infection, May–August 2022, in the southern Netherlands & Belgium (**a**) and the northern Netherlands (**b**). Dots indicate individual dead birds (jittered for clarity) that were ringed in their first year of life (1Y) in the respective study regions (marked by black squares). Dot colours represent the date of death, with blue shades indicating earlier deaths and green-yellow shades indicating later deaths. Three recoveries of dead birds ringed in the northern Netherlands are located outside the mapped extent: one in Ireland and two in the United Kingdom. Annual number of Sandwich terns ringed as 1Y (1995–2021) in the southern Netherlands & Belgium (**c**) and the northern Netherlands (**f**). Different grey shades represent different ring types (M = standard scientific metal ring, RM = field-readable or read metal ring, CR = colour-ring). **d** Region-specific apparent survival estimates (maximum likelihood estimates, back-transformed from the logit scale; 95% CI) of Sandwich terns ringed as 1Y (each an independently marked individual with its complete resighting history) across five age classes in the southern Netherlands & Belgium (**d**; *n* = 21,797 birds) and the northern Netherlands (**g**; *n* = 28,582 birds) (see also Supplementary Table 3). Estimated number of ringed birds alive per cohort in the southern Netherlands & Belgium (**e**) and the northern Netherlands (**h**) at the start of the HPAI outbreak in May 2022. Note that the y-axis scale differs by an order of magnitude with (**c**) and (**f**). Source data are provided as a Source Data file.

We assessed age-related mortality by first estimating pre-outbreak apparent survival rates (1995–2021, Fig. 1d, g) to reconstruct the expected age structure of the ringed population at the onset of the 2022 mass mortality event. For this, we used age-structured Cormack-Jolly-Seber (CJS) capture-mark-recapture models on resightings of 50,646 individuals ringed in their first year of life (1Y) in two regions that were at the core of the HPAI outbreak: the southern Netherlands & Belgium and the northern Netherlands (Fig. 1c, f). These survival estimates were used to derive the expected number of ringed birds alive in each region at the onset of the outbreak, which served as the denominator for the age-specific mortality analysis (Fig. 1e, h). During the outbreak, a total of 832 ringed birds of known age from the two study regions were recovered dead: 595 within the study regions (302 in the Southern Netherlands & Belgium and 293 in the Northern Netherlands) and 237 elsewhere (compared to a mean ± SD of 5.30 ± 3.36 ring recoveries per year in the preceding 27 years). We quantified HPAI-related mortality in two ways: (i) the ‘regional mortality’ estimate includes only birds recovered dead within their natal region which therefore corresponds most closely to the spatial structure of the CJS survival analysis, (ii) the ‘total mortality’ estimate includes all European recoveries of Dutch and Belgian ringed birds, both within and outside their natal region, providing a more comprehensive estimate of HPAI-related mortality by including birds that dispersed or started early migration following colony collapse and subsequently died elsewhere.

**Fig. 2:**
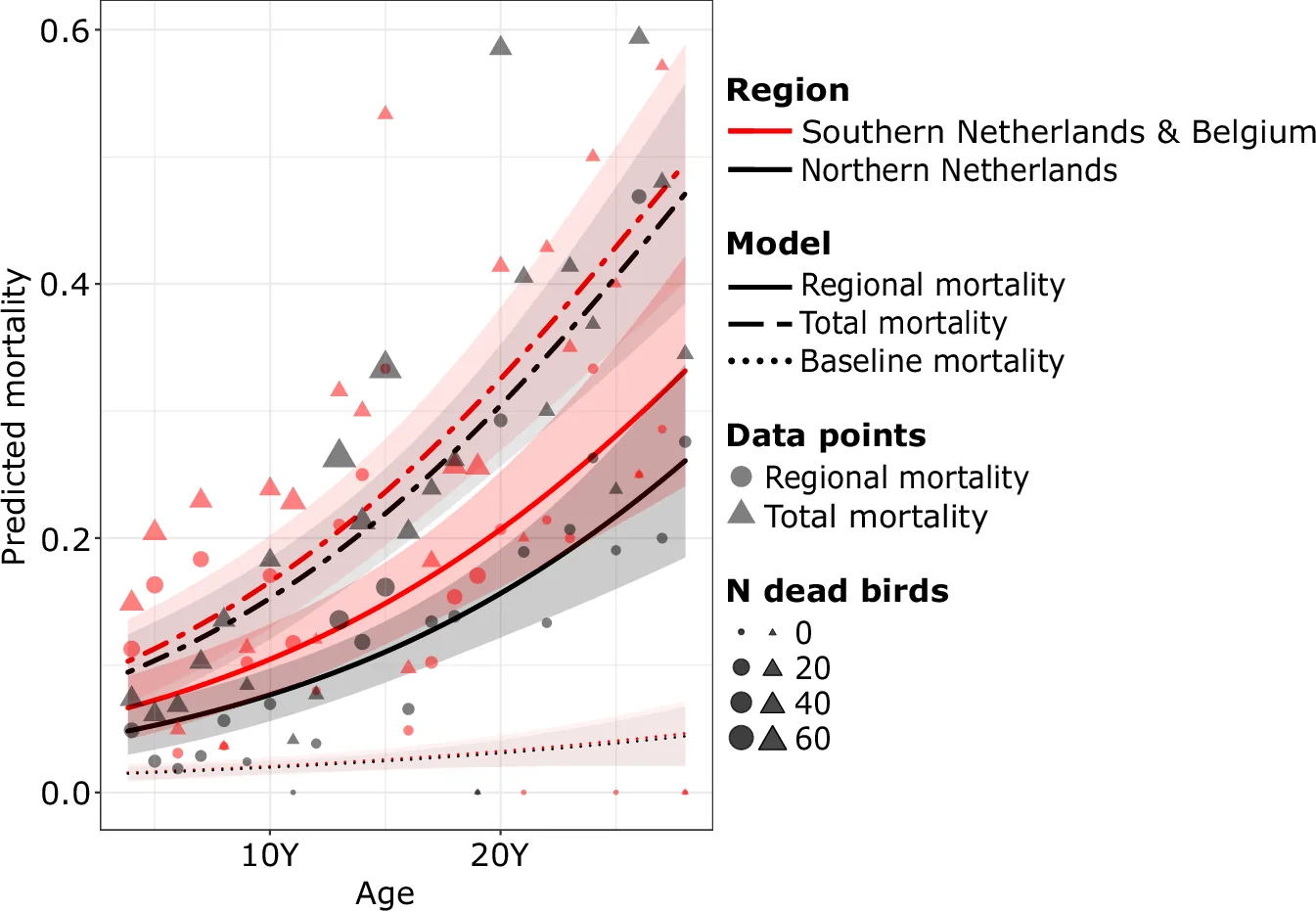
Predicted HPAI-related mortality of Sandwich terns during the 2022 breeding season (May–August) as a function of age and natal region. Data points represent the proportion of birds found dead in 2022 relative to the number of alive birds at the beginning of the HPAI-outbreak. Symbol size scales with the number of dead individuals per year of age. Two models were fitted: (i) a ‘regional mortality model’ including only birds recovered dead within their natal region (solid lines, dots), and (ii) a ‘total mortality model’ including all recoveries of Dutch and Belgian ringed birds in Europe, both in and outside their natal region (dashed lines, triangles). Predicted lines show the model-predicted mortality (back-transformed from the logit scale, i.e. the median predicted proportion) with 95% confidence intervals, estimated using a generalised linear mixed model (GLMM) with a beta-binomial error structure to account for overdispersion. Results are shown separately for the southern Netherlands & Belgium (black lines and symbols) and the northern Netherlands (red lines and symbols). Dotted lines indicate baseline mortality during June and July – the months in which 96.3% of HPAI-related casualties were recorded – and are included for reference. Birds up to and including their 3Y were excluded from the analysis, as most remain in the wintering areas or arrive late in the colonies. Birds over 28Y were also excluded because many were ringed with aluminium rather than steel rings, which are more likely to be lost and could therefore confound the analysis. Source data are provided as a Source Data file.

Generalised linear mixed models showed that predicted HPAI-related mortality increased significantly with age in both regions. Although both regional and total mortality of ringed birds was consistently higher in the southern Netherlands & Belgium, this geographic trend was not statistically significant (*P*_*regional*_ = 0.056, *P*_*total*_ = 0.54). Predicted HPAI-related mortality rates ranged from 6.7–10.5% in 4Y birds to 33.2–49.5% in 28Y birds in the southern Netherlands & Belgium, and from 4.9–9.6% to 26.1–47.1% in the northern Netherlands, respectively (Fig. 2). Each additional year of age was associated with an 8.4% increase in the odds of mortality in the regional mortality model (OR = 1.084, 95% CI: [1.056–1.112], *P* = 1.77 × 10⁻⁹) and a 9.3% increase in the total mortality model (OR = 1.093, 95% CI: [1.068–1.118], *P* = 4.89 ×  10⁻¹⁴). We repeated the analysis using the same demographic age classes as in the survival analysis instead of modelling age as a continuous predictor and this yielded consistent results: HPAI-related mortality probability increased with age and was highest in the oldest birds (Supplementary Table 4). The age effect was robust to uncertainty in the survival estimates. Across 4276 valid Monte Carlo iterations (85.5%; discarded iterations were concentrated in the oldest cohorts, where uncertainty compounds through successive years of survival estimation), the estimated age effect was always positive and remained a significant predictor of mortality in 99.9% of iterations for the regional mortality model (median *P*_*regional*_ = 3.2 × 10⁻⁹) and 99.9% of iterations for the total mortality model (median *P*_*total*_ = 2.4 × 10⁻¹³; Supplementary Fig. 1).

### Age-related arrival timing

To assess whether exposure to HPAI was influenced by age-related differences in arrival timing, we analysed first sightings of colour-ringed birds in the northern Netherlands, one of the hardest-hit regions in 2022 with well-monitored colonies. First-time breeders (4–5Y) arrived later than older birds (linear model, *β* ± SE = 23.63 ± 1.97, *t* = 11.98, *P* < 0.001), but still well before the onset of the outbreak (Supplementary Fig. 2). Among older birds (6–10Y), arrival timing did not vary with age (linear model, *β* ± SE = 0.43 ± 0.88, *t* = 0.49, *P* = 0.63), and median arrival dates (15–18 April) preceded the first detected cases by more than a month.

## Discussion

We show that HPAI-related mortality during the 2022 outbreak in northwestern European Sandwich terns was age-biased, with older individuals disproportionately affected, while mortality was not biased by sex. This pattern suggests that HPAI did not act as a random mortality source, but selectively removed individuals that likely contribute substantially to population growth. Below, we consider potential explanations for this age bias and its implications for recovery in long-lived seabirds.

Age- and sex-related mortality of seabirds has been documented in relation to fisheries bycatch^17^, migration^18^, wind farm collisions^19^, oil spills^20^, attraction to artificial lights^21^, food stress^22^ and environmental conditions^23^. These patterns are generally attributed to differences in dispersal strategies, at-sea distribution, foraging behaviour, or morphology. HPAI outbreaks affecting breeding Sandwich terns had not been documented prior to 2022. The large-scale epizootic affecting seabirds across northwestern Europe that year therefore represented the first such event, apart from mass mortality in great skuas (*Stercorarius skua*) reported in 2021^24^. Consequently, Sandwich tern populations are likely to have had little prior exposure to HPAI viruses and therefore limited population-level immunity. In 2022, all but one of the tested adults (*n* = 122) were confirmed positive for an HPAI H5N1 virus infection, and the timing of these cases coincided with the mass mortality events observed across Sandwich tern colonies in northwestern Europe. Together with the typically low baseline adult mortality during the breeding season in non-outbreak years (Fig. 2), this strongly suggests that the observed age-bias in mortality was primarily driven by the HPAI outbreak and may reflect differences in exposure or susceptibility, or both.

Arrival timing differed between first-time breeders and older birds, but all age classes were present in colonies well before the onset of the outbreak. This indicates that phenological differences in exposure are unlikely to explain the observed age bias in mortality. While first-time breeders tend to nest more in the periphery of a colony than older birds^25^, the high breeding densities of Sandwich terns, intense bird-to-bird contact and faecal transmission within colonies likely facilitated widespread exposure across age classes, making strong age-related differences in exposure due to within-colony nest location unlikely. Although age-related variation in breeding propensity could influence presence in colonies, available evidence from common tern (*Sterna hirundo*), suggests that breeding probability may actually decline with age^26^.

An alternative explanation for the pattern we found is age-related variation in susceptibility due to differences in physiological condition or immune function (immunosenescence), although evidence for this process in wild birds remains limited^27^. Experimental studies of HPAI H5 viruses in domestic waterbirds have in some cases reported lower mortality in older individuals^28^, and no age-related immune decline was detected in common terns^29^. These findings contrast with our results, highlighting that the mechanisms underlying age-biased mortality during HPAI outbreaks in wild seabirds remain poorly understood and warrant further investigation.

CJS models estimate apparent survival from natal-region resightings and therefore cannot disentangle permanent emigration from mortality. However, dispersal in Sandwich tern is distance-dependent, with most individuals recruiting to nearby colonies^30^. Modelling survival at large subregional scales likely captures much of this dispersal, reducing misclassification of emigration as mortality. To account for mortality occurring beyond the study area, we implemented a complementary total mortality model. Birds recovered outside the study regions were found later in the season than those recovered inside (Fig. 1a, b; median: 27 June outside, 15 June inside; Wilcoxon rank-sum test, W = 33,991, *P* < 2.2 × 10⁻¹⁶; see also ref. ^15^), consistent with earlier outbreak in the study areas and subsequent relocation following colony collapse or mate loss. Ring resightings confirm that Sandwich terns frequently relocated to foreign colonies – similar patterns were observed in northern gannets (*Morus bassanus*) following the 2022 HPAI outbreak^31^ –, with some even initiating a second breeding attempt^32^. While such movements may lead to underestimation of absolute mortality within regions, they are unlikely to generate systematic age-dependent bias. In addition, mortality during HPAI outbreaks in Sandwich terns was typically spatially clustered, with carcass detection rates generally high, enhancing detection and limiting recovery bias. Moreover, a substantial proportion of recoveries occurred on open beaches adjacent to breeding colonies, habitats used by both breeding adults and younger or non-breeding birds. This argues against age-dependent recovery bias as an explanation for the observed mortality patterns. Both models likely underestimate absolute mortality, since many birds that died, both within and outside the study regions, were probably not recovered or reported^15,33,34^. Integrating live encounters and recovery data within a multistate framework could further refine estimates of survival and recovery probabilities as additional data accumulate. More broadly, combining capture-mark-recapture data with serological information could yield valuable insights into patterns of exposure and susceptibility. Site-faithful, long-lived colonial species may thereby serve as effective sentinels of disease dynamics^35^.

The loss of older, more experienced, and potentially more productive individuals^25,36^ in this long-lived seabird species is concerning, but the long-term consequences for population recovery remain difficult to predict. Although instantaneous mortality during outbreaks in Dutch and Belgian colonies was estimated at 26.7% based on local dead bird recoveries (with true mortality likely to be higher^34,37^), the observed decline in breeding pairs in 2023 was smaller, at 21.4%. Recruitment of non-breeding adults (‘floaters’) and first-time breeders, together with immigration from other regions, may have temporarily buffered the short-term demographic impact of HPAI-related mortality^10,38^, suggesting a degree of demographic resilience. However, such compensatory processes may erode the pool of non-breeding individuals available to replace breeders lost during future mortality events. Concurrently, elevated population-level immunity may help mitigate the risk of future mass mortality events^8^. In addition, reduced densities of foraging terns around breeding colonies may increase higher per capita prey availability, potentially enhancing fecundity and facilitating population recovery^39^.

These uncertainties do not diminish the threat that HPAI poses to seabird populations worldwide, many of which have experienced severe mass mortality since 2022^40^. Seabirds – including penguins, albatrosses, petrels, gannets, gulls, terns and auks – share life-history traits such as delayed maturity, low annual reproductive output, and colonial breeding^41^, making them particularly vulnerable to adult mass mortality events caused by infectious diseases^42^. The loss of experienced individuals could reduce the reproductive output, delaying recovery, and increase the risk of long-term population declines. Quantifying these effects will require detailed data on age-specific survival and reproduction, and robust demographic modelling. Such changes may also erode genetic diversity and disrupt colony structure, potentially reducing the capacity of populations to respond to future environmental change. For seabirds already facing multiple pressures, including climate change, overfishing, predation, and habitat loss^43^, the emergence of HPAI adds an additional source of vulnerability in an increasingly unstable world.

## Methods

### Study species

Sandwich terns (*Thalasseus sandvicensis*) breed in dense ground colonies across northwestern Europe. In 2022, there were 67 colonies, with colony sizes varying widely (mean ± SD: 842 ± 1,422 breeding pairs, BP)^15^. Colonies can reach high densities^25^, with up to seven nests per m², such that a colony of 2000 BP may occupy as little as ~600 m².

The species is highly mobile, with frequent prospecting movements between colonies within a single breeding season^44,45^). High breeding densities, frequent direct contact between individuals, and the accumulation of faecal material around nests^46^ are likely to facilitate pathogen transmission within colonies, while frequent movements between colonies may promote spread at larger spatial scales.

### Study area and outbreak context

The study area comprised 12 colonies (11 in the Netherlands and one in Belgium), totaling 19,569 BP in 2022, within the northwestern European population of 59,632 BP^15^.

In typical years, adult mortality in breeding colonies is low. Between 1995 and 2021, 63 ringed Sandwich terns older than 3Y were recovered during the breeding season, the oldest being 19Y. Observations from an intensively monitored colony in the Dutch Delta (visited four to five times per week) between 2009 and 2018 (cumulative total 20,875 BP) recorded between zero and three dead adults per year, of which only four individuals were ringed. Observed causes of death included predation, collision, entanglement and egg binding.

In 2022, at least 39 of the 67 colonies were affected by HPAI^15^, resulting in the death of at least 19,326 adult birds, most between late May and early July. All colonies in the study area were affected, where 9815 adults were found dead, of which approximately 8200 were located within colonies^15,37^, indicating that this region formed the core of the outbreak in northwestern Europe.

Carcasses of adult Sandwich terns were tested for HPAI virus infection in several countries, including Belgium (*n *= 5, Sciensano), the Netherlands (*n* = 51, Wageningen Bioveterinary Research & Erasmus Institute), Germany (*n* = 54, regional veterinary diagnostic laboratories and Friedrich Loeffler Institut), Denmark (*n* = 6)^47^ and the United Kingdom (*n* = 6, The Animal and Plant Health Agency). All but one German bird tested positive.

### Ringing and resightings

Sandwich terns have been ringed continuously over multiple decades in the study area, primarily using scientific metal rings, with an extensive colour-ringing programme initiated in 2012. Between 1995 and 2020, an average ( ± SD) of 1,913 ± 850 chicks were ringed annually across 4–7 colonies.

Several characteristics of the species complicate resighting. Ring reading within colonies is difficult due to high breeding densities, with legs often obscured by neighbouring birds and vegetation. In addition, birds frequently take flight in response to human presence, further reducing opportunities for ring reading. Observations therefore rely primarily on mobile or fixed hides at colony edges. Additional resightings originate from opportunistic observations of colour-ringed individuals at coastal roosts, beaches and other sites outside breeding colonies.

### Collation of ringing data

We compiled metal ringing data of Sandwich terns covering the period between 1 May 1995 and 31 August 2022 from the national ringing schemes of Belgium and the Netherlands. Belgium hosts a single colony just across the Dutch border. Because this colony was abandoned in 2008 and there is substantial exchange between Belgium and the southern Netherlands, the data from both countries were grouped. The starting year was set at 1995, as from that year onward, all Belgian and Dutch birds were ringed with durable steel rings instead of the weaker aluminium ones, which are likely to be lost over time. In addition, we included data from a colour-ringing programme that started in 2012.

All data from the different sources were cross-checked for inconsistencies, such as birds being reported alive in one database but dead in another, conflicting dates and locations, or cases where birds were ‘resighted’ after being found dead. Incorrect records were corrected where possible and else removed. Ringing records from outside the focal period (May–July) or outside the study regions were also omitted, as were resightings from outside the study regions, because they conflict with the assumptions of the capture-mark-recapture models. Only birds ringed in their first year of life (indicated as 1Y, mostly birds ringed just before fledging in their natal colony) were retained as only for these birds exact age determination is possible. To minimize the effect of pre-fledging mortality on the survival estimates, we excluded chicks that died before the end of July in the year of ringing, but retained all other dead recoveries for the mortality predictions. Three types of rings were used during the study period: two types of scientific metal rings (standard rings (M) and larger, field-readable or read metal rings (RM)) and colour-rings (CR). In total, the final dataset contained more than 93,000 records: ringing data for 50,646 Sandwich terns ringed as 1Y (40,428 with M, 3136 with RM and 7082 with CR), 42,151 unique (excluding multiple observations of the same bird on the same day) live resightings (4,888 of M, 1027 of RM and 36,236 of CR), and 975 dead recoveries.

### Survival estimates

Mean annual apparent survival rates (*φ*, *Phi*) before the mass mortality event in 2022 (1995–2021) were estimated for the northern and the southern Netherlands (including Belgium) using Cormack-Jolly-Seber (CJS) models for encounter data. We used the *RMark* package (version 3.0.0)^48^ in *R* (version 4.5.1)^49^, which serves as an interface for the *MARK* program^50^. Our goal was to generate detailed age-specific survival estimates that reliably reflect survival trends across ages, while avoiding models that result in inestimable parameters for certain age classes.

To satisfy the assumptions of the CJS model, resightings were restricted to those within the natal region during a fixed recapture period (the breeding season, 1 May–31 July). Multiple resightings within the same year were pooled.

We used two approaches to evaluate the goodness-of-fit (GOF) of the mark-recapture data. First, we estimated the overdispersion coefficient (*ĉ*) using the parametric bootstrap GOF procedure in *MARK*, by dividing the observed deviance of the global model by the mean deviance from 100 bootstrap simulations. Second, we assessed the fit of the encounter histories to the CJS model using U-CARE^51^ via the *R2Ucare* package (version 1.0.2)^52^.

As is common with large and heterogeneous capture-mark-recapture datasets, most data subsets produced highly significant GOF test results, indicating violations of key CJS assumptions (Supplementary Table 1). These violations are partly due to the species’ ecology: after fledging, immature Sandwich terns remain at the wintering grounds and are essentially undetectable in the breeding areas, leading to inflated *χ²* values in the transience test (test3.sr). To account for this and to avoid overfitting, we defined a priori age-structured models with five age classes^53^. These reflect key life history stages: immatures (1–3Y), first-time breeders (4–5Y), young adults (6–10Y), medium-aged adults (11–15Y), and old adults (15 + Y).

We developed a series of models in which resighting probability was allowed to vary by age class, ring type (reflecting differences in ring readability, with resighting probability for M < RM < CR), and region, with and without interactions with time. Time was grouped into five-year intervals to reduce the risk of overfitting. Detection probability was also influenced by whether a bird was actively breeding in a given year and whether it nested in a colony (and at a nest site) where it could be observed. These factors likely contributed to the high *χ²* values observed in the trap-dependence test (test2.ct), indicating that resighting probabilities were not fully independent. To address this, we included a set of models that explicitly accounted for trap dependence^54^. For each of the model structures described above, we fitted a Pledger mixture model to account for unobserved individual heterogeneity in detection probability, potentially due to differences in sex, condition, or nest site^55^.

Overdispersion was accounted for in the calculation of standard errors and in model selection using Akaike’s Information Criterion adjusted for overdispersion (QAICc). Models with a ΔQAICc <2 were considered equally supported (Supplementary Table 2).

In principle, bias arising from uncertainty in age-specific survival estimates could be addressed using joint live-encounter and dead-recovery models^56^. However, the relatively small number of dead recoveries compared with the number of ringed individuals, combined with low resighting probabilities of immature birds (reflecting species ecology, with individuals remaining in wintering areas until 3–4Y), resulted in non-identifiable parameters, rendering this approach infeasible for the present dataset. Similarly, extending the models to include post-outbreak data to estimate temporal changes in survival before and after the outbreak also led to non-identifiable parameters.

### Age-composition of the ringed population

We multiplied the region- and age-specific apparent survival rates with the annual number of birds ringed (N_ringed_) in each region to calculate how many birds from each cohort (i.e., those born in the same year) were ‘estimated to be alive and present in the region’ in May 2022 (N_alive_). Specifically, we multiplied the annual survival estimates over the previous years for each cohort. For example, N_alive_ for birds in their 9th year of life (9Y) is calculated as:

$${{{{\rm{N}}}}}_{{{{\rm{alive}}}}/9{{{\rm{Y}}}}}={{{{\rm{N}}}}}_{{{{\rm{ringed}}}}}\times {{\varphi }_{1{{{\rm{Y}}}}-3{{{\rm{Y}}}}}}^{2}\times {{\varphi }_{4{{{\rm{Y}}}}-5{{{\rm{Y}}}}}}^{2}\times {{\varphi }_{6{{{\rm{Y}}}}-10{{{\rm{Y}}}}}}^{4}$$

and N_alive_ for birds in their 17Y as:

$${{{{\rm{N}}}}}_{{{{\rm{alive}}}}/17{{{\rm{Y}}}}}={{{{\rm{N}}}}}_{{{{\rm{ringed}}}}}\times {{\varphi }_{1{{{\rm{Y}}}}-3{{{\rm{Y}}}}}}^{2}\times {{\varphi }_{4{{{\rm{Y}}}}-5{{{\rm{Y}}}}}}^{2}\times {{\varphi }_{6{{{\rm{Y}}}}-10{{{\rm{Y}}}}}}^{5}\times {{\varphi }_{11-15{{{\rm{Y}}}}}}^{5}\times {{\varphi }_{15+{{{\rm{Y}}}}}}^{2}$$

This provided the age composition of the ringed population at the onset of the HPAI outbreak in May 2022.

### Age- and sex-composition of HPAI mortalities

Infection with HPAI virus often causes acute illness and inability to fly, leading to the discovery of numerous carcasses in or near breeding colonies^37^. In total, 832 birds in their 2Y or older, that were ringed as 1Y in Belgium or the Netherlands (allowing precise age determination), were recovered dead during the 2022 breeding season (May–August) and were presumed to have died from avian influenza. Of these, 595 were found within these two study regions. All dead individuals were identified using their scientific metal ring, which is present on all marked birds.

243 carcasses were collected and sexed between 29 May and 5 July 2022, 82 in the southern and 161 in the northern Netherlands.

### Arrival dates in the northern Netherlands

The dates of first sightings (15 March–15 July) of 419 Dutch colour-ringed Sandwich terns in their 3–10Y in the northern Netherlands were used to calculate the median arrival date per cohort. The colonies in the northern Netherlands were surveyed almost daily from mid-March in 2022.

### Statistical analyses

All statistical analyses were performed using *R* (v4.5.1)^49^. Birds up to their 3Y were excluded from the mortality analysis as 2Y old birds mostly remain in the wintering areas and the median arrival date of birds in their 3Y was 12 days later than the onset of the HPAI epidemic (see Supplementary Fig. 2). These birds therefore had a much lower probability of dying in the breeding areas. Birds older than 28Y were excluded because many were ringed with aluminium rather than steel rings, which are subject to rapid wear and subsequent ring loss and could therefore confound the analysis. For the 2022 HPAI outbreak, we modelled the number of dead birds in each cohort, together with the number estimated to be alive and present in the southern Netherlands & Belgium and the northern Netherlands at the start of the outbreak. These two counts were combined using the cbind() function and analysed using a generalised linear mixed model (GLMM) with a beta-binomial error distribution and a logit link to account for overdispersion, implemented in the *glmmTMB* package (v1.1.10)^57^. Fixed effects included age (covariate ranging from 4Y to 28Y), region (a two-level factor), with and without their interaction, while an observation-level random intercept was included to account for overdispersion. Model selection was based on AIC and likelihood ratio tests, and model fit was assessed using the *DHARMa* package (v0.4.7)^58^. To provide a baseline for comparison, we also modelled the theoretical number of birds expected to die under non-epidemic conditions by applying two months of expected mortality – derived from the annual survival estimates (100 - estimated survival) / (12 × 2) – to the estimated number of birds alive prior to the outbreak, and analysed these data using an identical model structure. To examine whether the observed age effect depended on how age was modelled, we also analysed age as a categorical variable based on the demographic age classes applied in the survival modelling. To assess whether the age effect was robust to uncertainty in the survival estimates underlying the number of birds presumed alive, we conducted a Monte Carlo sensitivity analysis (5,000 iterations). In each iteration, age- and region-specific survival probabilities were drawn simultaneously from a multivariate normal distribution on the logit scale, parameterised by the estimates and variance-covariance matrix of the CJS mixture model, and propagated through the cohort calculations to regenerate the number of birds presumed alive at the start of the HPAI outbreak in May 2022. Iterations yielding biologically impossible values (observed deaths exceeding estimated survivors in any cohort) were discarded. Each valid dataset was refitted with the regional and total mortality models above, and we recorded the proportion of iterations in which the age effect remained positive and significant.

To account for uncertainty in the spatial scope of mortality detection, we modelled predicted HPAI-related mortality in two ways. First, we included only dead birds recovered in their natal region where they were originally ringed, mirroring the structure of CJS models, where permanent emigration is not distinguished from mortality. This approach yields a conservative estimate of mortality (‘regional mortality’). Second, we used all dead recoveries of ringed birds regardless of where they were found, providing a more inclusive estimate of mortality (‘total mortality’).

We tested whether the sex ratio of adults found dead deviated from the expected 1:1 using a binomial test. Differences in age distributions between males and females were tested using a two-sided Wilcoxon rank-sum test. To assess the effect of age on the timing of first resighting, we fitted two linear regression models with the Julian day of first resighting as the dependent variable. In the first model, age was included as a five-level factor (6–10Y), and in the second as a two-level factor contrasting younger (4–5Y) and older (6–10Y) birds. We compared date of death between birds found inside and outside the study regions with a Wilcoxon rank-sum test, as both variables were non-normally distributed. Date values were converted to Julian days prior to analysis.

### Reporting summary

Further information on research design is available in the Nature Portfolio Reporting Summary linked to this article.

## Supplementary information

Supplementary Information

Reporting Summary

Transparent Peer Review file

## Source data

Source Data

## Data Availability

Datasets generated and/or analysed during the current study are appended as supplementary data. The data to reproduce the results of this study are also available on figshare (10.6084/m9.figshare.30478049). Source data are provided with this paper.

## Code availability

The code to reproduce the results of this study is available on figshare (10.6084/m9.figshare.30488306). We provide code implemented in *R* (v4.5.1).
