# Supplementary material for: Avian influenza amplified age-related mortality in a long-lived seabird
Source: Nat Commun. 2026 Aug 19;17:8653. doi: 10.1038/s41467-026-76726-7 (PMC13490437; doi:10.1038/s41467-026-76726-7)

Avian influenza amplified age-related mortality in a long-lived seabird

Supplementary Information

Supplementary Table 1 | Results of goodness-of-fit tests (the overall test and its component tests (TEST 3.SR, TEST 3.SM, TEST 2.CT, TEST 2.CL)) to assess whether the data fitted a time-dependent Cormack-Jolly-Seber (CJS) model, using the *R* package *R2ucare*. Each component test is a chi-square (χ^2) test, the overall GOF statistic is the sum of the component χ^2 statistics with pooled degrees of freedom. All tests are two-sided. No adjustment for multiple comparisons was applied. Exact *P*-values are given where computable, very small p-values are reported as $P < 2\times10^{-16}$. For the directional component tests (TEST 3.SR, TEST 2.CT), χ^2 is the squared directional statistic, df = degrees of freedom, $\hat{c}$ = overdispersion coefficient (the ratio χ^2/df) for the fully time-dependent global model, *P* = p-value.

Region	Ringtype	Test	χ^2	df	*P*	$\hat{c}$
Southern Netherlands & Belgium	metal	overall	96.3	50	$9.2\text{x}10^{-05}$	1.9
Southern Netherlands & Belgium	metal	test2.ct ('trap dependence')	26.8	12	0.008	2.2
Southern Netherlands & Belgium	metal	test2.cl	48.9	23	0.001	2.1
Southern Netherlands & Belgium	metal	test3.sr ('transience')	20.2	12	0.064	1.7
Southern Netherlands & Belgium	metal	test3.sm	0.5	3	0.927	0.2
Southern Netherlands & Belgium	colour	overall	945.5	47	$<2\text{x}10^{-16}$	20.1
Southern Netherlands & Belgium	colour	test2.ct ('trap dependence')	89.1	7	$<2\text{x}10^{-16}$	12.7
Southern Netherlands & Belgium	colour	test2.cl	86.6	21	$6.2\text{x}10^{-10}$	4.1
Southern Netherlands & Belgium	colour	test3.sr ('transience')	540.1	8	$<2\text{x}10^{-16}$	67.5
Southern Netherlands & Belgium	colour	test3.sm	229.6	11	$<2\text{x}10^{-16}$	20.9
Northern Netherlands	metal	overall	1455.8	153	$<2\text{x}10^{-16}$	9.5
Northern Netherlands	metal	test2.ct ('trap dependence')	111.9	24	$2.6\text{x}10^{-13}$	4.7
Northern Netherlands	metal	test2.cl	376.5	76	$<2\text{x}10^{-16}$	5.0
Northern Netherlands	metal	test3.sr ('transience')	829.0	24	$<2\text{x}10^{-16}$	34.5
Northern Netherlands	metal	test3.sm	138.4	29	$3.3\text{x}10^{-16}$	4.8
Northern Netherlands	colour	overall	1418.0	27	$<2\text{x}10^{-16}$	52.5
Northern Netherlands	colour	test2.ct ('trap dependence')	101.7	5	$<2\text{x}10^{-16}$	20.3
Northern Netherlands	colour	test2.cl	99.7	10	$<2\text{x}10^{-16}$	10.0
Northern Netherlands	colour	test3.sr ('transience')	615.9	5	$<2\text{x}10^{-16}$	123.2
Northern Netherlands	colour	test3.sm	600.7	7	$<2\text{x}10^{-16}$	85.8

Supplementary Table 2 | Model selection showing the 10 best survival (φ, *Phi*) and recapture (p) models. npar = number of estimated parameters, QAICc = quasi–Akaike Information Criterion corrected for overdispersion, ΔQAICc = difference in QAICc relative to the best-supported model, weight = QAICc weight representing the relative support for each model. AC = age class (1-3Y, 4-5Y, 6-10Y, 11-15Y, 15+Y), reg = region (southern Netherlands & Belgium and northern Netherlands), RT = ring type (metal, read metal and colour), td = trap dependence, mix = mixture model. Time was binned in 5 intervals (1995-2000-2005-2010-2015-2021) to avoid overfitting. The ĉ-value from the bootstrap GOF test (1.364), reflecting low residual overdispersion of the final model structure, was used to adjust standard errors and to compute QAICc.

φ	p	npar	QAICc	ΔQAICc	weight
**AC + reg**	**AC + RT + reg * time + td + mix**	25	27972	0	0.99
AC + reg	AC + RT * time + reg * time + td + mix	33	27981	9	0.01
AC + reg	AC * time + RT + reg * time + mix	39	27997	25	0.00
AC + reg	AC + RT * time + reg + td + mix	29	28018	46	0.00
AC + reg	AC * time + RT + reg + mix	35	28027	56	0.00
AC + reg	AC + RT + reg * time + mix	24	28084	112	0.00
AC + reg	AC + RT * time + reg * time + mix	32	28092	120	0.00
AC + reg	AC + RT * time + td + mix	28	28116	144	0.00
AC + reg	AC + RT * time + reg + mix	28	28121	150	0.00
AC + reg	AC * time + RT + mix	34	28325	354	0.00

Across all candidate models, survival (φ) was modelled as a function of age class and region – φ(AC + reg). Model selection focused on alternative structures for resighting probability (p). The best-supported model (weight = 0.99) included additive effects of age class and ring type, an interaction between region and time, as well as trap-dependence and a mixture component to account for individual heterogeneity – p(AC + RT + reg * time + td + mix). The second-ranked model, which additionally included an interaction between age class and region in p, received minimal support (weight = 0.01). All remaining models had ΔQAICc > 25 and negligible support. These results show that when survival was consistently modelled for age and regional differences, resighting probabilities were influenced by ring type (reflecting differences in readability of different ring types), temporal and regional interactions (reflecting observer effort changing over time, accessibility of colonies etc.), trap-dependence and individual heterogeneity.

Supplementary Table 3 | Estimates of apparent survival (φ) from the best model run in *RMark* (φ (age class + region) *p* (age class + ring type + region*time + td + mixture)) for five age classes for the northern Netherlands and southern Netherlands with Belgium. φ = apparent survival, se = standard error, lcl = lower 95% confidence limit, ucl = upper 95% confidence limit.

region	age class	φ	se	lcl	ucl
Southern Netherlands & Belgium	1–3Y	0.622	0.015	0.593	0.651
Southern Netherlands & Belgium	4–5Y	0.985	0.019	0.844	0.999
Southern Netherlands & Belgium	6–10Y	0.865	0.013	0.836	0.889
Southern Netherlands & Belgium	11–15Y	0.865	0.036	0.778	0.921
Southern Netherlands & Belgium	15+Y	0.808	0.029	0.745	0.858
Northern Netherlands	1–3Y	0.651	0.011	0.630	0.672
Northern Netherlands	4–5Y	0.987	0.017	0.858	0.999
Northern Netherlands	6–10Y	0.879	0.013	0.852	0.902
Northern Netherlands	11–15Y	0.879	0.031	0.805	0.927
Northern Netherlands	15+Y	0.827	0.025	0.772	0.870

Supplementary Table 4 | Results of a binomial generalized linear model (logit link, fitted by maximum likelihood using the *R* package *glmmTMB*) testing the effect of age class and region on HPAI-related mortality. The response variable was the number of birds found dead relative to the number of birds alive. The reference categories were birds aged 11–15Y and the Northern Netherlands. Coefficients are reported on the logit scale, *P*-values are from two-sided Wald z-tests and were not adjusted for multiple comparisons.

predictor	estimate	se	z	*P*
intercept	-1.222	0.076	-16.15	1.1×10^{-58}
4–5Y	-1.022	0.124	-8.27	1.4×10^{-16}
6–10Y	-0.870	0.110	-7.89	3.0×10^{-15}
15+Y	0.243	0.102	2.39	0.017
region	0.127	0.083	1.53	0.126

Supplementary Figure 1 | Monte Carlo sensitivity analysis of the effect of age on avian influenza mortality. Frequency distribution of *P*-values across 4,276 valid iterations (out of 5,000) for the regional mortality model (a) and total mortality model (b). Dashed lines indicate *P* = 0.05. Source data are provided as a Source Data file.

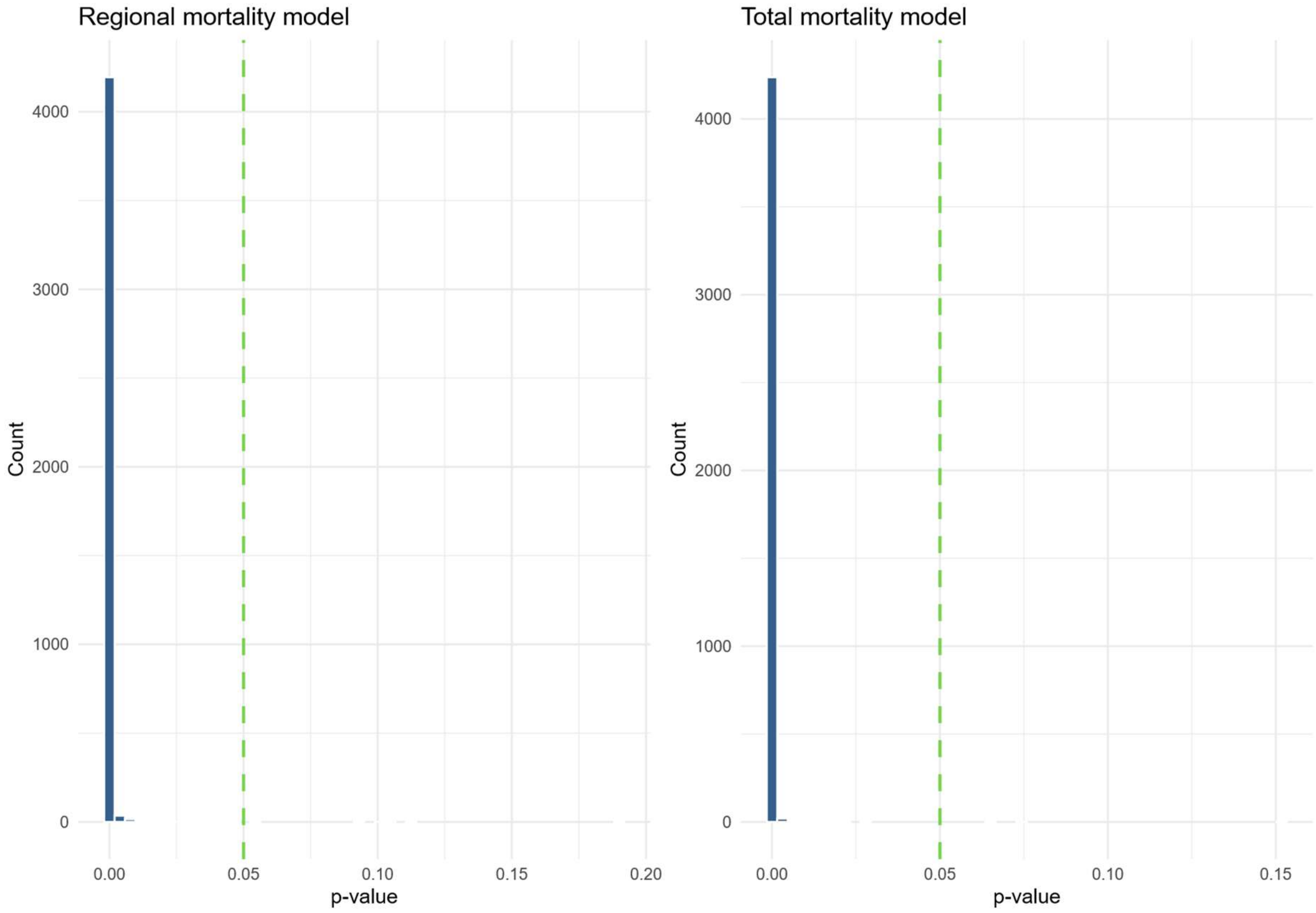

Supplementary Figure 2 | Median date of first sighting of Dutch colour-ringed Sandwich terns of known age in the northern Netherlands. Ridgeline plots show the density of observed birds per date for the age classes 3–10Y. The number of birds observed is shown on the right side of the graph. The blue arrow indicates the median arrival date of birds aged 6–10Y. The red arrow indicates the date when the first HPAI victim was found in the northern Netherlands. Source data are provided as a Source Data file.

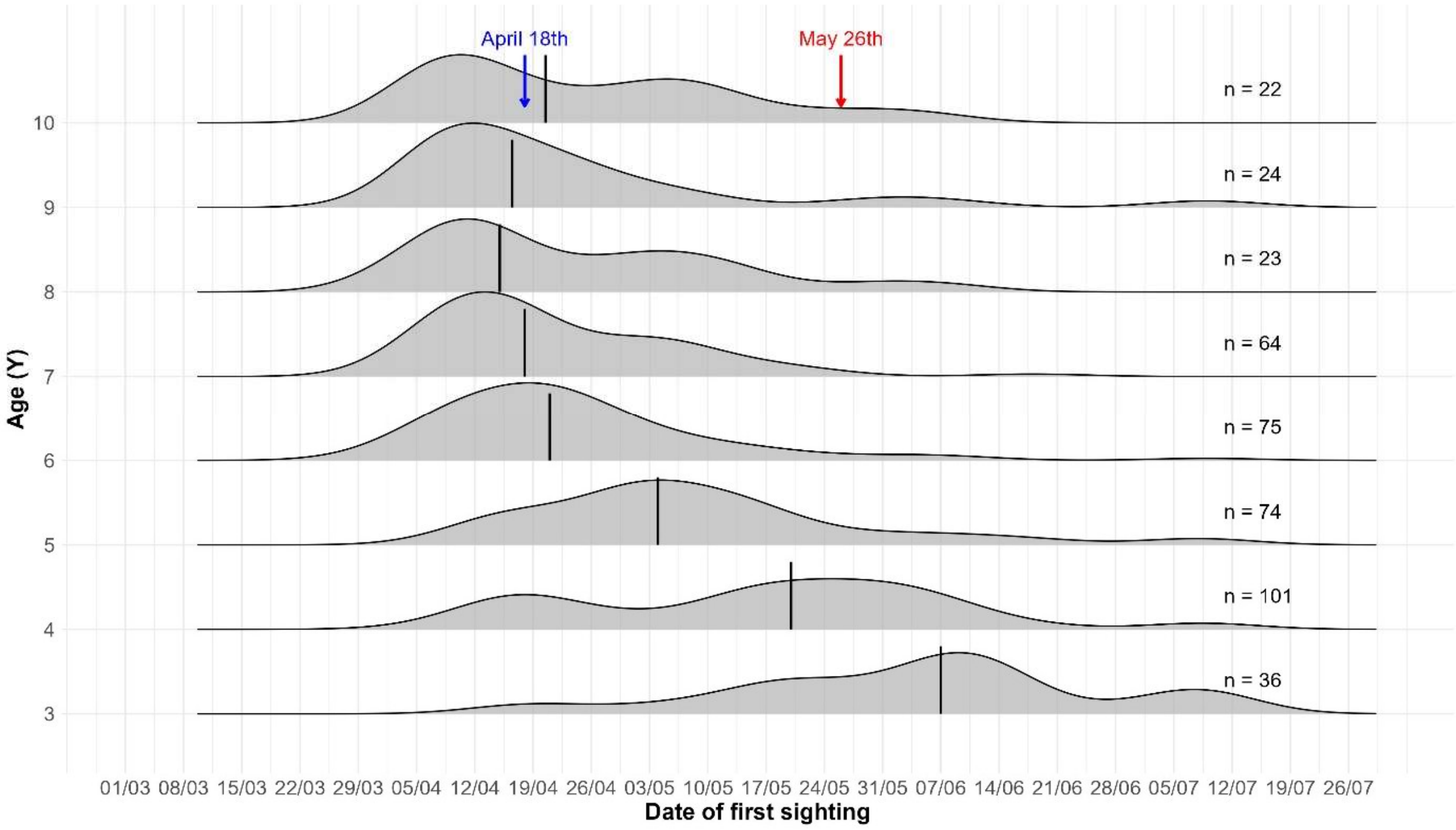

Supplement: Supplementary file 1 [file 41467_2026_76726_MOESM1_ESM.pdf]
