# Supplementary material for: Avian influenza amplified age-related mortality in a long-lived seabird
Source: Nat Commun. 2026 Aug 19;17:8653. doi: 10.1038/s41467-026-76726-7 (PMC13490437; doi:10.1038/s41467-026-76726-7)
Supplement: Supplementary file 2 [file 41467_2026_76726_MOESM2_ESM.pdf]

nature portfolio

Corresponding author(s): Ulrich Knief

Last updated by author(s): Jul 9, 2026

Reporting Summary

Nature Portfolio wishes to improve the reproducibility of the work that we publish. This form provides structure for consistency and transparency in reporting. For further information on Nature Portfolio policies, see our Editorial Policies and the Editorial Policy Checklist.

Statistics

For all statistical analyses, confirm that the following items are present in the figure legend, table legend, main text, or Methods section.

n/a	Confirmed	
☐	☒	The exact sample size (*n*) for each experimental group/condition, given as a discrete number and unit of measurement
☒	☐	A statement on whether measurements were taken from distinct samples or whether the same sample was measured repeatedly
☐	☒	The statistical test(s) used AND whether they are one- or two-sided<br>*Only common tests should be described solely by name; describe more complex techniques in the Methods section.*
☐	☒	A description of all covariates tested
☐	☒	A description of any assumptions or corrections, such as tests of normality and adjustment for multiple comparisons
☐	☒	A full description of the statistical parameters including central tendency (e.g. means) or other basic estimates (e.g. regression coefficient) AND variation (e.g. standard deviation) or associated estimates of uncertainty (e.g. confidence intervals)
☐	☒	For null hypothesis testing, the test statistic (e.g. *F*, *t*, *r*) with confidence intervals, effect sizes, degrees of freedom and *P* value noted<br>*Give P values as exact values whenever suitable.*
☒	☐	For Bayesian analysis, information on the choice of priors and Markov chain Monte Carlo settings
☒	☐	For hierarchical and complex designs, identification of the appropriate level for tests and full reporting of outcomes
☐	☒	Estimates of effect sizes (e.g. Cohen's *d*, Pearson's *r*), indicating how they were calculated

Our web collection on statistics for biologists contains articles on many of the points above.

Software and code

Policy information about availability of computer code

Data collection	No software was used to collect data for this study.
Data analysis	All analyses were conducted in R (v. 4.5.1) and Program Mark (v. 11.2).

For manuscripts utilizing custom algorithms or software that are central to the research but not yet described in published literature, software must be made available to editors and reviewers. We strongly encourage code deposition in a community repository (e.g. GitHub). See the Nature Portfolio guidelines for submitting code & software for further information.

Data

Policy information about availability of data

All manuscripts must include a data availability statement. This statement should provide the following information, where applicable:

- Accession codes, unique identifiers, or web links for publicly available datasets
- A description of any restrictions on data availability
- For clinical datasets or third party data, please ensure that the statement adheres to our policy

All data are available at https://doi.org/10.6084/m9.figshare.30478049. All code is available at https://doi.org/10.6084/m9.figshare.30488306.

Research involving human participants, their data, or biological material

Policy information about studies with human participants or human data. See also policy information about sex, gender (identity/presentation), and sexual orientation and race, ethnicity and racism.

Reporting on sex and gender	NA
Reporting on race, ethnicity, or other socially relevant groupings	NA
Population characteristics	NA
Recruitment	NA
Ethics oversight	NA

Note that full information on the approval of the study protocol must also be provided in the manuscript.

Field-specific reporting

Please select the one below that is the best fit for your research. If you are not sure, read the appropriate sections before making your selection.

☐ Life sciences ☐ Behavioural & social sciences ☒ Ecological, evolutionary & environmental sciences

For a reference copy of the document with all sections, see nature.com/documents/nr-reporting-summary-flat.pdf

Ecological, evolutionary & environmental sciences study design

All studies must disclose on these points even when the disclosure is negative.

Study description	We investigated age- and sex-specific mortality in Sandwich terns (Thalasseus sandvicensis) during the 2022 HPAI A (H5N1) clade 2.3.4.4b outbreak in northwestern Europe. Using individual-level ringing and resighting data collected over 28 years (1995–2022), we quantified mortality patterns and evaluated whether older or male/female birds were disproportionately affected.
Research sample	Our dataset comprised 50,646 Sandwich terns ringed in their first year of life (1Y) in the Netherlands and Belgium, supplemented with 42,151 live resightings and 975 dead recoveries. Sex was known for 243 adults collected during the outbreak.
Sampling strategy	All available data were used after validation.
Data collection	Ringing and resighting data were compiled from national ringing schemes and a colour-ringing program.
Timing and spatial scale	The study covers 1995–2022, with focus on May–August 2022 during the HPAI outbreak. Spatially, analyses were restricted to the southern Netherlands & Belgium and the northern Netherlands, which were at the epicentre of the outbreak.
Data exclusions	To satisfy the assumptions of the CJS model, resightings were restricted to those within the natal region during a fixed recapture period (the breeding season, 1 May – 31 July). For the GLMM analyses, birds younger than 4Y (which typically remain in the wintering areas or arrive late in the breeding season) and birds older than 28Y (due to known loss of old aluminium rings) were excluded to prevent bias in the models.
Reproducibility	This study did not involve experimental findings. All analyses were conducted in R (v4.5.1), using well-established packages for mark-recapture modeling (RMark, R2Ucare) and generalized linear mixed models (glmmTMB). To support reproducibility, the underlying data are available on Figshare (https://doi.org/10.6084/m9.figshare.30478049) and the analysis code is also accessible on Figshare (https://doi.org/10.6084/m9.figshare.30488306).
Randomization	Randomization was not relevant to this study as it was not an experimantal study and there was no assignment to groups.
Blinding	Data collection was conducted blind to the study hypothesis, and data handling and analyses followed standardized protocols to minimize observer bias.

Did the study involve field work? ☐ Yes ☒ No

Reporting for specific materials, systems and methods

We require information from authors about some types of materials, experimental systems and methods used in many studies. Here, indicate whether each material, system or method listed is relevant to your study. If you are not sure if a list item applies to your research, read the appropriate section before selecting a response.

Materials & experimental systems

n/a	Involved in the study
☒	☐ Antibodies
☒	☐ Eukaryotic cell lines
☒	☐ Palaeontology and archaeology
☒	☐ Animals and other organisms
☒	☐ Clinical data
☒	☐ Dual use research of concern
☒	☐ Plants

Methods

n/a	Involved in the study
☒	☐ ChIP-seq
☒	☐ Flow cytometry
☒	☐ MRI-based neuroimaging

Plants

Seed stocks	NA
Novel plant genotypes	NA
Authentication	NA
