# Supplementary material for: Avian influenza amplified age-related mortality in a long-lived seabird
Source: Nat Commun. 2026 Aug 19;17:8653. doi: 10.1038/s41467-026-76726-7 (PMC13490437; doi:10.1038/s41467-026-76726-7)
Supplement: Supplementary file 3 [file 41467_2026_76726_MOESM3_ESM.pdf]

nature portfolio

Peer Review File

Avian influenza amplified age-related mortality in a long-lived seabird

Corresponding Author: Dr Ulrich Knief

This file contains all reviewer reports in order by version, followed by all author rebuttals in order by version.

Version 0:

Reviewer comments:

Reviewer #1

(Remarks to the Author)
This manuscript reports important results on the demographic impact of high pathogenicity avian influenza (HPAI) in a wild bird species, the sandwich tern. That species, a seabird with a recorded lifespan of more than 30 years, was one of the bird species severely affected by HPAI in northwestern Europe, losing over 20 000 adults. Given the novelty of the impact of HPAI in many wild animal species, and the dramatic effects expected from field reports, such eco-epidemiological investigations are particularly important. The study is of an especially broad interest because of the results presented, but also because of the scientific approach used, which highlights the importance of long term monitoring in ecology and eco-epidemiology, and the importance of population ecology approaches in this context.
The study is based on the analyses of two rather unique data sets : first, long-term Capture-Mark-Recapture (CMR) data of live sandwich terns (1995-2021) collected in this species before the onset of the massive die-off due to HPAI in 2022, and second, recoveries of dead birds of known ages (i.e. individuals that had been ringed as chicks in the Netherlands and Belgium). As the authors report, during the outbreak, a total of 832 ringed birds of known age from the two study regions were recovered dead (595 in Belgium and the Netherlands and 237 elsewhere).
The manuscripts addresses the important question of potential age and sex effects of HPAI on the probability of dying in the considered species. For a sex effect, the answer is clear as the sex ratio of the sexed dead individuals recovered from the outbreak is 50/50 and no indication of a sex biases is present at any step of the study. Regarding the effect of age, a relatively ad hoc approach is used as it combines (i) the CMR data prior to the outbreak with (ii) data on the ages of the different marked individuals that were recovered dead at different times and locations once the outbreak started. The CMR data set is used to infer what proportion of each age class (and thus, numbers of individuals, when accounting for the number of individuals marked on their birth year in the different cohorts) would be expected to be alive at the time of the outbreak, so that it could be compared to that among the marked individuals recovered dead for each age class. The authors are carefull to consider as a potentially confounding effect on the risk of exposure the expected strong effect of age on the location of individuals at different time at the start of the breeding season, because older and more experienced individuals are well known in long lived colonial species to reach the breeding grounds and start breeding earlier than younger ones. As documented by the authors, this effect is nevertheless unkiley to have affected the results as the data presented show that all individuals of the relevant classes had reached the breeding areas once the outbreak occurred. Moreover, the analyses were carried out considering recovery data at different spatial scales, with similar overall results. The results suggest at strong effect of age on the probability of dying of HPAI over the outbreak, and this effect is expected to be due a mere age effect, not due to a higher exposure of older individuals to the virus at the breeding grounds. It is suggested that immunocenessence might be involved, but the main conclusion is that more work needs to be carried on this topic as very little is know about the role of immunity and age in bird-HPAI interactions in wild populations.
Overall, the manuscript is well written and the main data analyses and interpretation appear sound. The statistical analyses are advanced and make use of the different sources of information available, but it might be useful to discuss more at the end of the manuscript (1) some of the limits of the current analyses, and (2) potential improvements that could be made regarding the modelling, notably if some more data could be available. CMR statistical modelling as applied in the study allowed the authors to account for various sources of uncertainty, notably those affecting detection probabilities of live birds of different age classes and their capture histories in the period prior to the outbreak, but after the onset of the outbreak, only dead-recovery data are used to infer an effect of HPAI on survival as a function of age. Beyond the two-step ad hoc modelling approach used (that uses simple products of annual survival estimates for the series of age classes to compute the proportions of individuals still alive for each age class; see formulas lines 264 and 268), would the use of recapture/resighting data after the onset of the outbreak be useful to account for potential biases ? In particular, this

approach may lead to an especially high sensitivity of the modelling outcome to the estimation of annual survival in the older age classes, without accounting for uncertainty in the estimates. If, for a reason or another, the annual survival rates estimated for an age class of older individuals were overestimated with the CMR modelling prior to the outbreak, if I am correct, it could lead to artificially increase an effect of age on the apparent proportions of dead individuals in cohorts of old individuals. If enough data could be available for the years after the outbreak, some of these issues could be addressed using a joint modelling of live-encounter and dead-recovery data before, during and after the outbreak year using e.g. a multistate CMR model to estimate survival, live resighting and dead-recovery probabilities (Burnham 1992, Badia-Boher et al. 2026). In relation to that, it may also be useful to mention the potential opportunity, in the future and/or in other comparable systems, to also use resighting/recapture data gathered before but also after the outbreak with information on the serological status of live individuals as a source of information about their history of exposure to the virus. Of course, this may be difficult due to the scarcity of high quality data in most species, but site-faithfull long-lived colonial species monitored at their breeding sites could provide ideal species for that (e.g., Gamble et al. 2020). Identifying whether individuals of different ages have been exposed and survived differentially could then be powerfully determined by using CMR modelling before and after the outbreak if one could have access to the serological status of individuals.
Overall the manuscript is very well written, with a carreful use of terms. On Line 123, I nevertheless wonder if it would not be more proper to replace 'migration' with 'dispersal', as it relates to the fact that individuals moved between two sites where they bred, not seasonal migration.

Cited references:
Badia-Boher, J.A. et al. 2026. Evaluating the demographic impacts of the highly pathogenic avian influenza panzootic. Journal of Applied Ecology 63: e70234.
Burnham, K.P. 1993. A theory for combined analysis of ring recovery and recapture data. In J. D. Lebreton & P. M. North (Eds.), Marked individuals in the study of bird population (pp. 199–213). Birkhäuser Verlag.
Gamble et al. 2020. Next-generation serology: integrating cross-sectional and capture–recapture approaches to infer disease dynamics. Ecology 101: e02923.

(Remarks on code availability)
The code as provided in the submitted files appears usable to reproduce the study (presence of a detailed README file).

Reviewer #2

(Remarks to the Author)
This manuscript examines whether mortality associated with the 2022 highly pathogenic avian influenza (HPAI) outbreak in northwestern Europe was selective with respect to age or sex in Sandwich terns, using long-term individual-level ringing data combined with the number of dead recoveries during a key outbreak year. The authors report no evidence for sex-biased mortality and a strong positive association between age and mortality risk, suggesting that older individuals were disproportionately affected. The question is timely and important given ongoing concern about the demographic impacts of HPAI on long-lived seabird populations.

Overall, the study is carefully assembled and clearly presented. However, several aspects of the interpretation would benefit from more careful framing and additional context.

The finding that older Sandwich terns were more likely to die during an acute outbreak raises some red flags - not because it is implausible, but because it runs counter to well-established expectations. In long-lived species, older individuals are typically those that have survived multiple selective filters and are often considered relatively robust. When results contradict prior biological intuition, the burden of proof shifts: the evidence needs to be especially solid, and alternative explanations need to be actively explored and ruled out.

A key issue is attribution of cause of death. All recovered birds are presumed to have died from HPAI, but it is unclear what proportion of carcasses were tested or confirmed positive, how representative those tested birds are of the broader mortality dataset, or whether other potential causes of death were excluded. Background mortality during the breeding season, typical causes of death in Sandwich tern colonies, and whether these causes are known to show age structure are not described. Without this context, it is difficult to assess how much of the observed age pattern reflects HPAI-specific mortality versus all-cause mortality occurring during an HPAI outbreak. While HPAI was clearly widespread in these colonies, it remains possible - even if unlikely - that an alternative process or more than one process contributed to mortality during this period. Given experience in wildlife disease systems where large mortality events initially assumed to be attributed to a single pathogen later proved to have mixed or alternative causes, this uncertainty should be acknowledged more explicitly, ideally with supporting data to allow readers to better evaluate the inference.

Related to this, the interpretation would benefit from clearer separation between exposure and susceptibility. A plausible explanation for the observed age pattern is increased exposure in older birds rather than increased intrinsic vulnerability. Older birds may be more likely to arrive earlier at breeding colonies, occupy central nesting positions, interact more frequently with conspecifics, and engage more intensively in incubation and brooding, all of which could increase contact with infectious material in a colony experiencing an outbreak. These are reasonable hypotheses, but they represent exposure mechanisms rather than age-related differences in disease susceptibility. The manuscript would benefit from a more explicit discussion of this distinction, including whether and how Sandwich terns are known to exhibit these types of age-stratified behavioral patterns, and from clearer acknowledgement that the current data cannot disentangle these alternatives.

The age-specific mortality estimates also rely on assumptions that deserve more explicit treatment. The analysis assumes that recovery probability does not vary systematically with age. In a colony-based outbreak, this assumption may not hold if older birds are more likely to be present in monitored areas, closer to observers, or at times when carcass detection is highest. While the authors distinguish between regional and total recovery estimates, discussion of how sensitive the age effect is to plausible age-related differences in recovery probability would strengthen confidence in the conclusions.

Similarly, the denominators used to estimate age-specific mortality are derived from apparent survival estimates based on natal-region resightings. Apparent survival conflates mortality with permanent emigration, and age-related differences in site fidelity or movement could influence estimated age composition at the onset of the outbreak. I am not a specialist in capture–mark–recapture or survival modelling, and evaluation of the technical details of these models would benefit from review by someone with specific expertise in this area. From a general perspective, however, it would be helpful for the authors to clarify how robust the age effect is to alternative assumptions or to analyses restricted to subsets of the data with particularly high resighting probability.

The treatment of age in the mortality analyses also warrants clarification. Age is modelled as a linear covariate, while the underlying age structure used in the survival modelling is based on discrete life-history stages. While these age categories appear to be well justified from a demographic perspective, their relevance to infection risk or disease severity during an HPAI outbreak is less clear. It is not obvious that mortality risk from an acute viral infection should increase with each additional year of age. The manuscript would be strengthened by either providing a clearer biological justification for a linear age effect in this context, or demonstrating that the observed pattern is robust to alternative age structures (e.g., age classes or nonlinear terms).

Relatedly, the manuscript would benefit from additional context on what is known about historical avian influenza exposure (whether LPAIV or HPAIV) in Sandwich terns and in these colonies specifically. Baseline levels of exposure and immunity in this system are not indicated. Without such information, interpretations linking age directly to vulnerability should be framed cautiously and clearly distinguished from life-history-based demographic interpretations.

One additional point concerns ring type. Ring type affects resighting probability and may also influence dead recovery reporting, and it is correlated with age. Including ring type explicitly in the mortality models, or demonstrating that the age effect persists within a homogeneous ring subset, would help rule out potential confounding.

Finally, the current analyses treat sex and age largely as separate questions. Given the biological plausibility that sex differences could vary across age classes (and vice versa), it would be useful to explore whether there is evidence for an age × sex interaction in mortality risk, at least within the subset of carcasses for which sex is known.

Provided these methodological and interpretive considerations are addressed, this study has the potential to make a meaningful contribution to understanding the demographic consequences of HPAI in long-lived seabirds.

(Remarks on code availability)

Reviewer #3

(Remarks to the Author)
I appreciate the opportunity to read and review the manuscript, ‘Avian influenza causes age-related mortality in a long-lived seabird’ recently submitted to Nature Communications. In this manuscript, the authors assess sex and age-related mortality relative to a well-documented highly pathogenic avian influenza (HPAI)-associated mortality event in Sandwich Terns breeding in Belgium and the Netherlands. The authors found no evidence for sex-related mortality. In contrast but that predicted mortality positively correlated with age. The authors draw comparisons to survival estimates made for birds during a 1995-2021 pre-outbreak period.

Generally speaking, I thought the premise of the paper to be well-founded and the approach to be fairly rigorous. However, aspects of the summary and interpretation gave me some pause. Several examples are provided below.

First, the authors frame their results as HPAI causing age-related mortality (i.e., the manuscript title); however, as I infer from their pre-outbreak modelling results, Sandwich Terns naturally experience age-related mortality (i.e., lines 60-61; Figure 1d and 1g). Therefore, I am less sure that HPAI is causing this trend rather than influencing the magnitude thereof. If this understanding is correctly, I feel that it is important to frame results accordingly. Furthermore, the authors go on to draw comparison between pre-outbreak Sandwich Tern survival and predicted mortality rates co-incident with the 2022 mortality event (i.e., lines 75-86). However, I found these comparisons to be less-than-straightforward. For example, as I understood from the authors own words (lines 68-74), that it is probably methodologically most appropriate to compare the pre-outbreak survival estimates to the ‘regional’ mortality rate; however, this does not stop the authors from also drawing parallels to their calculated ‘total’ mortality rate. Considering only the former (i.e., more appropriate) comparison, it’s not entirely clear to me that estimated pre-outbreak mortality of 4Y birds (Figure 1d and 1g) was appreciably lower as compared to the regional mortality rate (Figure 2) given uncertainty. The same holds true for older birds as well, though apparent differences in methodology makes direct comparisons challenging. This is all to state that the authors’ case that ‘HPAI causes age-related mortality’ seems to me, unclear at best, and poorly supported at worst. I tend to believe the former, but improved conveyance

of information is certainly warranted.

Additionally, the authors explore whether phenology contributed to age-related differences in mortality (lines 103-111; lines 285-288); however, I found the logic for and execution of this exploratory analysis somewhat confusing. As best I can infer, the analytical framework was not particularly robust and somewhat reliant on assumptions regarding the arrival of birds of different age classes. Again, this indicates another place in the manuscript where the narrative could be improved.

Additional, and relatively minor, examples of somewhat questionable interpretation are sprinkled elsewhere throughout the narrative. For example, line 40 references the mortality of 'millions' of (presumably) seabirds on account of HPAI, but the cited reference provides no such empirical evidence. Lines 117-119 refers to the finding that HPAI disproportionally affected more experienced and potentially more productive birds, though no metrics of breeding performance were incorporated into this investigation. Lines 122-124 highlights how recruitment of new breeders may mask demographic effects, but there is no mention of how recruitment may also indicate species resiliency.

On the positive side, I think the authors are addressing an important issue and I applaud their work to improve our understanding of HPAI induced mortality among migratory birds. On the less-positive side, I personally feel that this manuscript would benefit from some considerable revision to improve rigor of analyses, comparison of information, and the clarity of messages conveyed.

(Remarks on code availability)

Version 1:

Reviewer comments:

Reviewer #1

(Remarks to the Author)
This is a well written manuscript on an important and timely topic. The authors have done a nice work at taking into account of the comments made by the reviewers notaby of expanding the discussion on exposure versus susceptibility issue as noted by reviewer 2.

Nevertheless, I find that there may still be a critical issue with the ad hoc approach used. The unexpected finding of a high apparent death rate of older individuals compared to younger ones following the HPAI outbreak is based on the use of impressive data sets and extensive modelling accounting for sources of variability in detectability of marked individuals, but one aspect that seems to have been overlooked, and which could be weakening strongly the strenght of the inference, is related to a key assumption made to obtain the denominator values to compute the rate of mortalities of the different age groups. More specifically, the estimations of the annual survival rate for each age class are very important for calculating the denominator values (see the multiplication formulas on lines 361-375 – some are at the power 5), but they have been obtained using a CMR modelling analysis that assumed no time variability in annual survival rates (see table 2 of the supplement with all the compared models: the CMR modelling is done on the years before the HPAI outbreak year 2022 and only age class [AC] and region [Reg] are considered as factors potentially associated with the annual survival, not [time]). I do not think that this is a fair assumption because many factors varying in time (and space/time) could affect the annual survival of different age class of individuals. For instance, the authors relax this assumption when they do their ad hoc approach because for the year 2022 they consider that the survival rate may have been dramatically lower in 2022 (due to HPAI) and different among age classes, and that is actually their main results. It would thus be important to explore (or at least clearly discuss) how potential temporal variability in annual survival rates in the years before 2022 could have affected the predicted numbers of alive individuals in the different age classes in 2022, and thus potentially the main result (for instance, what could have been the effect of a hypothetical high survival for the young age classes and low survival for the old age classes in 2020 and 2021. At present, the very high annual survival rate estimates [>0.985] for the 4-5 year old individuals obtained with the constant time for phi modelling approach may certainly explain a low expected negative effect of HPAI on the young adult individuals because this leads to a large denominator for this age group for 2022). I realize that the authors may have simplified the set of models considered for the sake of tractability, but a solution may be to explore the sensitivity of the result via a formal sensitivity analysis (considering different scenarios of values for the annual survival rates of each age class prior to 2022); this could permit to explore convincigly the robustness of the result.

Minor remarks :
The citation suggested for discussing serology and CMR approaches was Gamble et al. 2020 'Next-generation serology: integrating cross-sectional and capture–recapture approaches to infer disease dynamics' Ecology 101: e02923', not Gamble et al. 2020 in 'J Appl Ecol'.

(Remarks on code availability)

Reviewer #2

(Remarks to the Author)

(Remarks on code availability)

Reviewer #3

(Remarks to the Author)
I appreciate the authors addressing my concerns in their response to reviews.

(Remarks on code availability)

Version 2:

Reviewer comments:

Reviewer #1

(Remarks to the Author)
Thank you very much for taking into account of the comments made on the last round of reviews, notably by incorportating the sensitivity analyses. I am please to recommend this nice manuscript for publication.

(Remarks on code availability)

Open Access This Peer Review File is licensed under a Creative Commons Attribution 4.0 International License, which permits use, sharing, adaptation, distribution and reproduction in any medium or format, as long as you give appropriate credit to the original author(s) and the source, provide a link to the Creative Commons license, and indicate if changes were made.
In cases where reviewers are anonymous, credit should be given to 'Anonymous Referee' and the source.
The images or other third party material in this Peer Review File are included in the article's Creative Commons license, unless indicated otherwise in a credit line to the material. If material is not included in the article's Creative Commons license and your intended use is not permitted by statutory regulation or exceeds the permitted use, you will need to obtain permission directly from the copyright holder.
To view a copy of this license, visit https://creativecommons.org/licenses/by/4.0/

Response to Reviewers

We thank the reviewers for their careful evaluation of our manuscript and for their constructive and insightful comments. These suggestions have helped us improve the clarity and robustness of the manuscript. We have revised the manuscript accordingly and provide detailed, point-by-point responses below. For clarity, line numbers cited in the responses refer to the clean version of the manuscript after all track changes have been accepted

Reviewer #1 (Remarks to the Author):

Comment 1.1: This manuscript reports important results on the demographic impact of high pathogenicity avian influenza (HPAI) in a wild bird species, the Sandwich tern… Overall, the manuscript is well written and the main data analyses and interpretation appear sound.

Author response: *We thank the reviewer for the positive assessment of the manuscript and for the constructive comments below.*

Comment 1.2: It might be useful to discuss more at the end of the manuscript (1) some of the limits of the current analyses, and (2) potential improvements that could be made regarding the modelling, notably if some more data could be available.

Author response: *We thank the reviewer for this helpful suggestion. We have expanded the Discussion section to more explicitly acknowledge the limitations of the current analyses and to outline potential improvements to the modelling framework if additional data become available (lines 144–149 & 163–167).*

Comment 1.3: After the onset of the outbreak, only dead-recovery data are used to infer an effect of HPAI on survival as a function of age. Beyond the two-step ad hoc modelling approach used, would the use of recapture/resighting data after the onset of the outbreak be useful to account for potential biases?

Author response: *We thank the reviewer for this helpful suggestion. Incorporating recapture/resighting data after the onset of the outbreak would indeed be a valuable addition. However, post-outbreak recapture/resighting data are currently limited, partly due to the high mortality among ringed birds during the outbreak. As a result, including the available post-outbreak data led to inestimable parameters, reflecting the limited sample size rather than biological patterns. We therefore opted for the more robust two-step approach based on dead-recovery data, but we acknowledge that integrating post-outbreak resighting data would be an important improvement when more data become available. We discuss this now in lines 163–164 and 353–359.*

Comment 1.4: In particular, this approach may lead to an especially high sensitivity of the modelling outcome to the estimation of annual survival in the older age classes, without accounting for uncertainty in the estimates. If, for a reason or another, the annual survival rates estimated for an age class of older individuals were overestimated with the CMR modelling prior to the outbreak, if I am correct,

it could lead to artificially increase an effect of age on the apparent proportions of dead individuals in cohorts of old individuals.

Author response: *We thank the reviewer for raising this important point regarding potential bias in the estimation of age-specific survival. Importantly, the bias raised by the reviewer would arise from underestimation (not overestimation) of survival in older age classes, which could inflate the apparent age effect.*

As suggested by the reviewer, such potential bias could in principle be addressed using joint live-encounter/dead-recovery models (e.g. Burnham 1993). We explored such models, but even the simplest formulations resulted in inestimable parameters. This reflects several characteristics of the Sandwich tern dataset. Dead recoveries of birds >1Y are very rare (143 recoveries from >50,000 ringed individuals between May 1995 and May 2022; mean 5.3 ± 3.4 per year), providing little additional information while increasing the number of parameters relative to a Cormack-Jolly-Seber model. In addition, most immatures remain in wintering areas during their 2Y and 3Y resulting in very low resighting probabilities in the breeding areas. Finally, Sandwich terns breed in dense, difficult-to-access colonies and show high mobility between colonies even within breeding seasons, further complicating resighting probabilities. To mitigate these issues, we used a CJS model incorporating two broad regions (including sightings outside colonies), multiple age classes, correction for trap dependence and a finite-mixture structure to account for heterogeneity in resighting probability. To clarify this point, we have added a paragraph in the Methods section explaining why joint live-encounter and dead-recovery models were not feasible for this dataset (lines 353–359).

Importantly, the observed decline in survival with age is consistent with well-documented senescence patterns in long-lived seabirds (e.g. common guillemot, wandering albatross, black-legged kittiwake, herring gull). In the well-studied common tern, another long-lived tern species, adult survival follows a convex trajectory, declining from 0.93 at age 2 to 0.73 by age 25 (Zhang et al. *2015, https://doi.org/10.1111/1365-2656.12259), and similar survival patterns have been reported in atlantic puffins (Landsem* et al. *2023, https://doi.org/10.1111/1365-2656.13884).*

Comment 1.5: If enough data could be available for the years after the outbreak, some of these issues could be addressed using a joint modelling of live-encounter and dead-recovery data before, during and after the outbreak year using e.g. a multistate CMR model to estimate survival, live resighting and dead-recovery probabilities (Burnham 1992, Badia-Boher et al. 2026).

Author response: *We agree and have added a sentence in the manuscript noting that joint modelling of live encounters and dead recoveries before, during, and after the outbreak would be a valuable extension once sufficient post-outbreak data become available (lines 163–164). Our approach differs from that of Badia-Boher* et al. *(2026) in that our objective is to estimate survival across detailed age classes rather than changes in overall adult survival. In addition, the ratio of dead recoveries to ringed individuals is substantially lower in our dataset, which limits the feasibility and statistical power of such joint models.*

Comment 1.6: In relation to that, it may also be useful to mention the potential opportunity, in the future and/or in other comparable systems, to also use resighting/recapture data gathered before but

also after the outbreak with information on the serological status of live individuals as a source of information about their history of exposure to the virus. Of course, this may be difficult due to the scarcity of high quality data in most species, but site-faithfull long-lived colonial species monitored at their breeding sites could provide ideal species for that (e.g., Gamble et al. 2020). Identifying whether individuals of different ages have been exposed and survived differentially could then be powerfully determined by using CMR modelling before and after the outbreak if one could have access to the serological status of individuals.

Author response: *We agree that incorporating serological status into CMR analyses could provide valuable insights into age-specific exposure and survival following HPAI outbreaks, as suggested by the reviewer. However, such approaches are most feasible in highly site-faithful species or in systems where previously infected individuals show identifiable external markers, as recently documented in northern gannets (Lane* et al. *2023, https://doi.org/10.1111/ibi.13275; Lewis* et al. *2026, https://doi.org/10.1111/ibi.13449). Implementing this framework would also require extensive ringing to ensure adequate sample sizes across age classes. Given the low site fidelity and very high breeding densities of Sandwich terns, obtaining such data would be extremely challenging. We now highlight the potential value of this approach for future studies in the manuscript (lines 164–167).*

Comment 1.7: On Line 123, I nevertheless wonder if it would not be more proper to replace 'migration' with 'dispersal', as it relates to the fact that individuals moved between two sites where they bred, not seasonal migration.

Author response: *We agree and rephrased the sentence, it now reads 'Recruitment of non-breeding adults ('floaters') and first-time breeders, together with immigration from other regions, may have temporarily buffered the short-term demographic impact of HPAI-related mortality, suggesting a degree of demographic resilience.' (lines 172–175).*

Reviewer #2 (Remarks to the Author):

Comment 2.1: Overall, the study is carefully assembled and clearly presented. However, several aspects of the interpretation would benefit from more careful framing and additional context.

Author response: *We thank the reviewer for the positive assessment of the manuscript. We have thoroughly revised the manuscript to provide additional context and to clarify the framing of several interpretations and results where needed.*

Comment 2.2: The finding that older Sandwich terns were more likely to die during an acute outbreak raises some red flags - not because it is implausible, but because it runs counter to well-established expectations. In long-lived species, older individuals are typically those that have survived multiple selective filters and are often considered relatively robust. When results contradict prior biological intuition, the burden of proof shifts: the evidence needs to be especially solid, and alternative explanations need to be actively explored and ruled out.

Author response: *We agree that special care is needed when interpreting this somewhat counter-intuitive result, and we appreciate the reviewer's point that older individuals in long-lived species are often considered high-quality survivors. However, this expectation mainly applies to mortality from chronic environmental pressures and established pathogens, whereas mortality patterns during outbreaks of novel pathogens can differ substantially. When a new virus enters a population, prior selective filtering for pathogen resistance has not occurred, and age-related susceptibility may instead be driven by other factors. Similar age-related mortality patterns during (avian) influenza outbreaks have been documented in humans and other species (e.g. Dudley & MacKay 2013, http://dx.doi.org/10.1016/j.jcv.2013.09.004; Kang & In Jung 2020, https://doi.org/10.3947/ic.2020.52.2.154; O'Driscoll* et al. *2021, http://dx.doi.org/10.1038/s41586-020-2918-0; Cairns* et al. *2025, https://doi.org/10.18103/mra.v13i1.6186). We therefore consider the observed pattern biologically plausible, although we agree that such results require careful evaluation of alternative explanations. These are addressed in the following sections.*

Comment 2.3: A key issue is attribution of cause of death. All recovered birds are presumed to have died from HPAI, but it is unclear what proportion of carcasses were tested or confirmed positive, how representative those tested birds are of the broader mortality dataset, or whether other potential causes of death were excluded. Background mortality during the breeding season, typical causes of death in Sandwich tern colonies, and whether these causes are known to show age structure are not described. Without this context, it is difficult to assess how much of the observed age pattern reflects HPAI-specific mortality versus all-cause mortality occurring during an HPAI outbreak. While HPAI was clearly widespread in these colonies, it remains possible - even if unlikely - that an alternative process or more than one process contributed to mortality during this period. Given experience in wildlife disease systems where large mortality events initially assumed to be attributed to a single pathogen later proved to have mixed or alternative causes, this uncertainty should be acknowledged more explicitly, ideally with supporting data to allow readers to better evaluate the inference.

Author response: *We thank the reviewer for raising this important point and agree that the manuscript could be improved on this point. The scale and timing of mortality, coinciding with confirmed HPAI outbreaks across multiple colonies, make alternative explanations for the observed mortality pattern highly*

unlikely. To clarify attribution of mortality, we have expanded the Methods section with additional information on (1) key ecological characteristics of the study species (lines 250–257) and (2) the characteristics of the 2022 HPAI outbreak in Sandwich terns, including typical background mortality during the breeding season and results of the tested adults.

Across the affected colonies, adults were tested for HPAI in the Netherlands (n = 51), Belgium (n = 5), Germany (n = 54), the UK (n = 6) and Denmark (n = 6), all but one German bird were positive. These data are now reported in the manuscript (lines 122–124 & 272–276). Background adult mortality in Sandwich Tern colonies during the breeding season is very low. In a colony of approximately 3,000 breeding pairs, typically only 0–3 dead adults are found over an entire season. Between 1995 and 2021, 63 ringed Sandwich Terns older than 3Y were recovered during the breeding season, the oldest birds to be recovered were 19Y. We now report this in the manuscript (lines 124–125 and 262–267). We also added a reference line to Fig. 2 indicating a two-month baseline mortality (96.4% of HPAI casualties were found in June and July).

Comment 2.4: Related to this, the interpretation would benefit from clearer separation between exposure and susceptibility. A plausible explanation for the observed age pattern is increased exposure in older birds rather than increased intrinsic vulnerability. Older birds may be more likely to arrive earlier at breeding colonies, occupy central nesting positions, interact more frequently with conspecifics, and engage more intensively in incubation and brooding, all of which could increase contact with infectious material in a colony experiencing an outbreak. These are reasonable hypotheses, but they represent exposure mechanisms rather than age-related differences in disease susceptibility. The manuscript would benefit from a more explicit discussion of this distinction, including whether and how Sandwich terns are known to exhibit these types of age-stratified behavioral patterns, and from clearer acknowledgement that the current data cannot disentangle these alternatives.

Author response: *We thank the reviewer for highlighting the necessity to more clearly separate the differences in pathogen exposure and intrinsic susceptibility. We have thoroughly revised the Discussion to more clearly separate these mechanisms. Exposure mechanisms now have a separate paragraph (lines 128–136) discussing phenology, position in the colony and breeding propensity. Likewise, susceptibility is discussed in a separate paragraph. We also added a sentence acknowledging that the mechanisms underlying age-biased mortality during HPAI outbreaks in wild seabirds remain poorly understood and warrant further investigation (lines 137–143).*

Comment 2.5: The age-specific mortality estimates also rely on assumptions that deserve more explicit treatment. The analysis assumes that recovery probability does not vary systematically with age. In a colony-based outbreak, this assumption may not hold if older birds are more likely to be present in monitored areas, closer to observers, or at times when carcass detection is highest. While the authors distinguish between regional and total recovery estimates, discussion of how sensitive the age effect is to plausible age-related differences in recovery probability would strengthen confidence in the conclusions.

Author response: *We agree with the reviewer that age-related variation in recovery probability could influence mortality estimates. In Sandwich Tern, as in many long-lived species, first-time breeders (4–5Y) are known to recruit incompletely and are therefore less likely to be present in the colony, which*

could reduce their probability of recovery. However, empirical data on age-specific breeding propensity among older individuals are lacking. Evidence from a well-studied colony of common tern of known-age indicates that breeding propensity is high at 5Y and may decline again at older (from ~13Y) ages (Zhang et al. *2015, https://doi.org/10.1111/1365-2656.12259). Importantly, Sandwich Tern breed in very dense colonies on small, open substrates, where mortality during HPAI outbreaks is highly localized and carcass detection is generally high. Such structure has a strongly positive effect on detection probability, so in that respect no bias is to be expected. Additionally, many observations are made on the open beaches near the colony, where young birds and possible non-breeding adults are also likely to spend time. We have clarified this assumption and its potential implications in the revised manuscript (lines 156–161).*

Comment 2.6: Similarly, the denominators used to estimate age-specific mortality are derived from apparent survival estimates based on natal-region resightings. Apparent survival conflates mortality with permanent emigration, and age-related differences in site fidelity or movement could influence estimated age composition at the onset of the outbreak. I am not a specialist in capture-mark-recapture or survival modelling, and evaluation of the technical details of these models would benefit from review by someone with specific expertise in this area. From a general perspective, however, it would be helpful for the authors to clarify how robust the age effect is to alternative assumptions or to analyses restricted to subsets of the data with particularly high resighting probability.

Author response: *We thank the reviewer for raising this important point. As Sandwich Terns are highly mobile and frequently move between colonies and subregions, the distinction between mortality and permanent emigration is indeed an important consideration when interpreting apparent survival estimates. However, dispersal in this species is distance-dependent, with most individuals recruiting to colonies relatively close to their natal sites and often within the same broader subregion. To minimise potential bias from permanent emigration, we structured the survival analyses at the level of two large subregions rather than individual colonies. Estimating survival at the colony level resulted in inestimable parameters due to sparse data, whereas modelling at the subregional scale allowed us to incorporate resightings of birds breeding outside their natal colony. This approach therefore captures many movements between colonies and reduces the risk that emigration is misinterpreted as mortality in the apparent survival estimates. We have clarified this point in the Discussion when describing the limitations of the capture-mark-recapture analysis (lines 144–148).*

Comment 2.7: The treatment of age in the mortality analyses also warrants clarification. Age is modelled as a linear covariate, while the underlying age structure used in the survival modelling is based on discrete life-history stages. While these age categories appear to be well justified from a demographic perspective, their relevance to infection risk or disease severity during an HPAI outbreak is less clear. It is not obvious that mortality risk from an acute viral infection should increase with each additional year of age. The manuscript would be strengthened by either providing a clearer biological justification for a linear age effect in this context, or demonstrating that the observed pattern is robust to alternative age structures (e.g., age classes or nonlinear terms).

Author response: *We thank the reviewer for raising this point. In the original analysis, age was included as a continuous covariate to test for a general trend in mortality across the adult age range. However,*

we agree that the relationship between age and mortality during an acute viral outbreak may not necessarily follow a strictly linear pattern. To assess the robustness of our results, we repeated the analysis using age as a categorical variable based on the demographic age classes applied in the survival modelling. This analysis yielded the same qualitative pattern, with mortality increasing markedly in the older age classes during the outbreak. We have added this clarification to the Methods (lines 411–413) and note the robustness of the age effect to alternative age structures in the manuscript (lines 94–97).

Comment 2.8: Relatedly, the manuscript would benefit from additional context on what is known about historical avian influenza exposure (whether LPAIV or HPAIV) in Sandwich terns and in these colonies specifically. Baseline levels of exposure and immunity in this system are not indicated. Without such information, interpretations linking age directly to vulnerability should be framed cautiously and clearly distinguished from life-history-based demographic interpretations.

Author response: *We thank the reviewer for this helpful suggestion and have added additional context on historical avian influenza exposure in Sandwich Tern (lines 117–122) and distinguish exposure and susceptibility mechanisms more clearly now (see also Comment 2.4).*

Available evidence suggests that prior exposure of Sandwich Tern to avian influenza viruses is extremely limited. Apart from a single reported AIV-positive individual (Sinnecker et al. *1982, cited in Stallknecht & Brown 2008, https://doi.org/10.1002/9780813818634), there are, to our knowledge, no studies documenting LPAIV prevalence or HPAIV outbreaks in breeding colonies before 2022. More broadly, the 2022 HPAI epizootic represented the first large-scale outbreak affecting breeding seabird colonies in northwestern Europe, with the notable exception of mortality events in Great Skua in 2021 (Banyard* et al. *2022, https://doi.org/10.3390/v14020212). Consequently, Sandwich Tern populations are likely to have had little prior exposure to HPAI viruses, and therefore limited population-level immunity. Evidence from other tern species remains sparse but broadly consistent with this interpretation, indicating historically low exposure and a recent increase in antibody prevalence following the 2022 outbreak (Knief* et al. *2026, https://doi.org/10.1101/2025.06.18.660293).*

Comment 2.9: One additional point concerns ring type. Ring type affects resighting probability and may also influence dead recovery reporting, and it is correlated with age. Including ring type explicitly in the mortality models, or demonstrating that the age effect persists within a homogeneous ring subset, would help rule out potential confounding.

Author response: *We thank the reviewer for raising this important and thoughtful point. In our dataset, all dead recoveries were identified using the metal ring, which is present on every marked individual. Colour rings are primarily used to facilitate resightings of live individuals and therefore mainly affect encounter probabilities in the CMR analyses, rather than the identification of dead individuals. We agree that colour rings could, in principle, increase the likelihood of detection during a cursory check. However, standard recovery protocols involve a sufficiently careful inspection of carcasses, such that the metal ring should be detected regardless of the presence or absence of colour rings. We therefore consider it unlikely that recovery probability differs systematically with ring type. Nevertheless, we acknowledge the reviewer's concern and have clarified this point in the manuscript (lines 382–383).*

Comment 2.10: Finally, the current analyses treat sex and age largely as separate questions. Given the biological plausibility that sex differences could vary across age classes (and vice versa), it would be useful to explore whether there is evidence for an age × sex interaction in mortality risk, at least within the subset of carcasses for which sex is known.

Author response: *We thank the reviewer for this helpful suggestion and agree that exploring a potential age × sex interaction in mortality risk would be biologically informative. However, such an analysis would require information on the sex-specific number of individuals alive in each age class at the start of the outbreak. While sex was determined for a subset of recovered carcasses, the sex of the individuals estimated to be alive within each cohort is largely unknown. Consequently, we are unable to estimate sex-specific mortality rates or formally test an age × sex interaction within the GLMM framework used in our study.*

To nevertheless address the reviewer's point as far as possible with the available data, we examined whether the subset of carcasses with known sex showed any evidence of sex-specific age structure. Specifically, we compared the age distributions of males and females using a two-sided Wilcoxon rank-sum test. We found no evidence for a difference in age distributions between sexes ($W = 4412.5$, $P = 0.33$), suggesting that males and females were similarly represented across age classes among recovered individuals.

We acknowledge that this does not fully resolve the question of a potential interaction, but it provides some reassurance that strong sex-specific age biases are unlikely in the recovered sample. We have now clarified this in the Methods (lines 421–422) and included a brief discussion in the manuscript (lines 64–66).

Reviewer #3 (Remarks to the Author):

Comment 3.1: Generally speaking, I thought the premise of the paper to be well-founded and the approach to be fairly rigorous.

Author response: *We thank the reviewer for this positive assessment of the study and its methodological approach.*

Comment 3.2: First, the authors frame their results as HPAI causing age-related mortality (i.e., the manuscript title); however, as I infer from their pre-outbreak modelling results, Sandwich Terns naturally experience age-related mortality (i.e., lines 60-61; Figure 1d and 1g). Therefore, I am less sure that HPAI is causing this trend rather than influencing the magnitude thereof. If this understanding is correctly, I feel that it is important to frame results accordingly.

Author response: *We thank the reviewer for this insightful comment. We agree that HPAI appears to have amplified an underlying pattern of age-related mortality in Sandwich Tern rather than caused it. We have therefore revised the title and rephrased the relevant sections of the manuscript to clarify that the outbreak increased the magnitude of age-dependent mortality, rather than introducing the pattern itself.*

Comment 3.3: Furthermore, the authors go on to draw comparison between pre-outbreak Sandwich Tern survival and predicted mortality rates co-incident with the 2022 mortality event (i.e., lines 75-86). However, I found these comparisons to be less-than-straightforward. For example, as I understood from the authors own words (lines 68-74), that it is probably methodologically most appropriate to compare the pre-outbreak survival estimates to the 'regional' mortality rate; however, this does not stop the authors from also drawing parallels to their calculated 'total' mortality rate. Considering only the former (i.e., more appropriate) comparison, it's not entirely clear to me that estimated pre-outbreak mortality of 4Y birds (Figure 1d and 1g) was appreciably lower as compared to the regional mortality rate (Figure 2) given uncertainty. The same holds true for older birds as well, though apparent differences in methodology makes direct comparisons challenging. This is all to state that the authors' case that 'HPAI causes age-related mortality' seems to me, unclear at best, and poorly supported at worst. I tend to believe the former, but improved conveyance of information is certainly warranted.

Author response: *We thank the reviewer for highlighting this point and agree that the original wording could give the impression that pre-outbreak survival estimates were directly compared with outbreak mortality estimates. This was not our intention, and we have revised the manuscript to clarify this (lines 69–76). The pre-outbreak survival estimates were used solely to estimate the number of birds expected to be alive at the onset of the outbreak, which served as the denominator for calculating mortality proportions during the HPAI event. As the survival estimates were derived from a Cormack-Jolly-Seber model based on resightings within natal regions, the 'regional' mortality estimate represents the most methodologically consistent comparison with the survival analysis. This regional estimate includes only birds recovered dead within their natal region, whereas the 'total' mortality estimate additionally includes individuals recovered outside their natal region, for example birds that may have dispersed or started early migration following colony collapse and subsequently died elsewhere. We have therefore*

clarified the distinction between these estimates and the rationale for presenting both in the revised manuscript (lines 80–85).

Comment 3.4: Additionally, the authors explore whether phenology contributed to age-related differences in mortality (lines 103-111; lines 285-288); however, I found the logic for and execution of this exploratory analysis somewhat confusing. As best I can infer, the analytical framework was not particularly robust and somewhat reliant on assumptions regarding the arrival of birds of different age classes. Again, this indicates another place in the manuscript where the narrative could be improved.

Author response: *We thank the reviewer for highlighting this point and agree that the rationale and interpretation of this exploratory analysis were not sufficiently clear in the original manuscript. We have therefore substantially revised the relevant sections to improve both the narrative and the transparency of the underlying assumptions.*

In the revised manuscript, we now more clearly distinguish between exposure (lines 128–136) and susceptibility (lines 137–141) and as two non-mutually exclusive mechanisms that could generate the observed age-related mortality pattern, and we explicitly acknowledge the difficulty of disentangling these processes (lines 141–143). We present the phenology analysis more explicitly as an exploratory assessment of whether age-related differences in arrival timing could plausibly contribute to variation in exposure during the outbreak, rather than as a definitive test. We also clarify the biological reasoning underlying this hypothesis. We have revised the text (lines 100–106 & 128-130) to more clearly describe the analytical framework, its assumptions, and its limitations, and to ensure that the exploratory nature of this analysis is appropriately conveyed.

Comment 3.5: Line 40 references the mortality of 'millions'… but the cited reference provides no such empirical evidence.

Author response: *We thank the reviewer for pointing this out. The original wording indeed overstated the evidence provided by the cited reference. We have therefore revised the sentence to read 'with total mortality in wild birds at least 1.4 million individuals', which more accurately reflects the available data (line 42).*

Comment 3.6: Lines 117–119 refers to more experienced and potentially more productive birds, though no metrics of breeding performance were incorporated into this investigation.

Author response: *We thank the reviewer for this helpful comment. We agree that age-specific breeding performance was not quantified in Sandwich Tern within the scope of this study. The term 'potentially' was used deliberately, as increases in breeding performance with age have been documented in several well-studied long-lived seabirds. For example, in a well-monitored colony of common terns of known age, breeding productivity increases with age up to approximately 13Y before levelling off in older individuals (Zhang* et al. *2015, https://doi.org/10.1111/1365-2656.12259). To our knowledge, the only available study in Sandwich tern (Veen 1977, https://www.jstor.org/stable/30039178) similarly reports higher breeding success in individuals aged ≥5Y compared to younger birds. We have now added both references to support the statement (line 168).*

Comment 3.7: Lines 122–124 highlights how recruitment of new breeders may mask demographic effects, but there is no mention of how recruitment may also indicate species resiliency.

Author response: *We thank the reviewer for this insightful observation and agree that increased recruitment may also reflect demographic resilience following a mortality event. At the same time, elevated recruitment may reduce the pool of non-breeding individuals (both floaters and first-time breeders), potentially diminishing the population's buffering capacity against future mortality events. We have revised the relevant sentence in the manuscript to explicitly acknowledge both interpretations (lines 172–176).*

Response to Reviewers

We thank the reviewers for their careful evaluation of the revised manuscript and for their additional constructive and insightful comments. We have revised the manuscript accordingly and provide detailed, point-by-point responses below. For clarity, line numbers cited in the responses refer to the clean version of the manuscript after all track changes have been accepted.

REVIEWER COMMENTS

Reviewer #1 (Remarks to the Author):

Comment 1.1: This is a well written manuscript on an important and timely topic. The authors have done a nice work at taking into account of the comments made by the reviewers notaby of expanding the discussion on exposure versus susceptibility issue as noted by reviewer 2.

Author response: *We thank the reviewer for the positive assessment and for recognising the revisions made in the previous round. We address the remaining comments below. For clarity, we have grouped our responses slightly differently from the order of the original comment, beginning with the sensitivity analysis (the main addition to the manuscript) and then addressing the supporting points.*

Comment 1.2: I realize that the authors may have simplified the set of models considered for the sake of tractability, but a solution may be to explore the sensitivity of the result via a formal sensitivity analysis (considering different scenarios of values for the annual survival rates of each age class prior to 2022); this could permit to explore convincigly the robustness of the result.

Author response: *We thank the reviewer for stressing the importance of using trustworthy survival estimates in the calculation of the number of birds presumed alive, and for the suggestion of a sensitivity analysis. We very much agree this strengthens the analysis and the manuscript.*
We conducted a Monte Carlo sensitivity analysis (n = 5,000 iterations). In each iteration, age- and region-specific survival probabilities were sampled simultaneously from a multivariate normal distribution on the logit scale, using the parameter estimates and variance-covariance matrix from the CJS model. Because the number of birds presumed alive is obtained by multiplying successive annual survival probabilities, uncertainty in the survival estimates propagates multiplicatively through these calculations. The resulting accumulation of uncertainty is greatest in the oldest age classes, where estimates depend on the longest sequence of annual survival probabilities. Consequently, biologically impossible estimates (observed HPAI deaths exceeding estimated birds presumed alive in any cohort) were concentrated in the oldest cohorts (ages 24–28), where occasional extreme draws produced near-zero population estimates incompatible with observed mortality. Such iterations were discarded, leaving 4,276 valid iterations (85.58%). In rare instances (< 0.05% of iterations), extreme tail draws from the survival distribution produced impossible estimates across a broader range of ages.
Across all valid iterations, the effect of age on avian influenza mortality remained positive. The regional mortality model was significant in 99.9% of iterations (median $P = 3.2 \times 10^{-9}$) and the total mortality model in 99.9% of iterations (median $P = 2.4 \times 10^{-13}$), confirming that the finding is robust to uncertainty in survival estimation.
We have expanded the Results section (lines 97-102) and the Methods section (lines 418-427) to include the sensitivity analysis.

Comment 1.3: It would thus be important to explore (or at least clearly discuss) how potential temporal variability in annual survival rates in the years before 2022 could have affected the predicted numbers of alive individuals in the different age classes in 2022, and thus potentially the main result (for instance, what could have been the effect of a hypothetical high survival for the young age classes and low survival for the old age classes in 2020 and 2021.

Author response: *We note that a hypothetical situation with higher survival in young age classes and lower survival in old age classes before 2022 would in fact only strengthen the age effect: higher survival inflates the number of young birds presumed alive (lowering their apparent mortality), while lower survival reduces the number of old birds (raising theirs). To provide a conservative test of the robustness of our results, we therefore explored the opposite scenario, in which survival was reduced in younger age classes and increased in older age classes. Specifically, from 2017 to 2022, the yearly survival of the five youngest ages (4Y–8Y) was set 10% lower than the mean CJS estimates, and the yearly survival of the five oldest ages (24Y–28Y) 10% higher. With the exception of the 4–5Y age class, whose adjusted values remained within its comparatively wide 95% confidence interval, these modifications placed the adjusted survival values 3–6 percentage points beyond the relevant confidence limit of the original estimates (below the lower bound for the young ages and above the upper bound for the old ages) and therefore represent a deliberately conservative sensitivity analysis. Even under this conservative scenario, the age effect remained highly significant in both the regional ($P = 6.90 \times 10^{-5}$) and total mortality models ($P = 6.88 \times 10^{-7}$).*

Comment 1.4: The CMR modelling is done on the years before the HPAI outbreak year 2022 and only age class [AC] and region [Reg] are considered as factors potentially associated with the annual survival, not [time]). I do not think that this is a fair assumption because many factors varying in time (and space/time) could affect the annual survival of different age class of individuals. For instance, the authors relax this assumption when they do their ad hoc approach because for the year 2022 they consider that the survival rate may have been dramatically lower in 2022 (due to HPAI) and different among age classes, and that is actually their main results.

Author response: *We did not include time as a factor associated with annual survival because models incorporating even a simple time term (survival per decade) returned multiple inestimable parameters, making them effectively unusable for our purpose. Beyond this technical constraint, what we assume absent before 2022 is age-specific temporal variation (year-to-year fluctuations that selectively affect particular age classes) not age structure itself. Because the wintering quarters span most of the West African coast and birds of all ages intermingle there, an ordinary mortality source would not selectively depress survival on a large scale in particular age classes. Nor do we see evidence of sudden higher or lower mortality during the breeding seasons up to 2022. Most Sandwich tern colonies in the study area are well monitored, so a sudden increase in dead birds would have been noted. The apparent increasing susceptibility of Sandwich terns to ordinary causes of death as they age is already incorporated into the mortality estimates.*

What distinguishes 2022 from previous years is precisely a mortality shock of the kind absent in ordinary years: a novel pathogen, against which the birds had no antibodies, entered the population and caused a mass mortality event. This differs fundamentally from ordinary causes such as food shortage, hunting pressure, or botulism, which are spatially limited and which most birds can escape.

Also, the elevated 2022 mortality is not a survival parameter that we assumed or fitted, it enters the analysis as a directly observed quantity. The numerator is the number of ringed birds reported dead

from HPAI during the outbreak (832 individuals of known age for the study population), and the denominator is the number of ringed birds presumed alive at the start of the outbreak, derived from the CJS estimates. The CJS model therefore supplies only the pre-2022 denominators, it is not used to infer a time-varying survival rate for 2022 itself.

Comment 1.5: At present, the very high annual survival rate estimates [>0.985] for the 4-5 year old individuals obtained with the constant time for phi modelling approach may certainly explain a low expected negative effect of HPAI on the young adult individuals because this leads to a large denominator for this age group for 2022).

Author response: *While the survival estimates for 4–5Y birds are indeed high, they are supported by the underlying resighting data and are not solely a consequence of the modelling. The CJS framework estimates these survival probabilities after explicitly accounting for imperfect detection, and the raw resighting data are consistent with the result: in the 2012 colour-ringed cohort, of 204 birds resighted at 3Y or later, 195 (95.6%) were resighted at 4Y or later and 184 of those 195 (94.4%) at 5Y or later, with the 2013 cohort showing the same pattern (96.8% and 95.9%). Because these proportions combine survival with resighting probability, we do not interpret them as survival rates directly. Rather, their high and only gradually declining values are consistent with the high apparent survival estimated by the model. Consequently, the large number of birds presumed alive in these age classes is driven by the empirical resighting data and the resulting survival estimates, rather than by an unrealistic modelling assumption.*

Comment 1.6: The citation suggested for discussing serology and CMR approaches was Gamble et al. 2020 'Next-generation serology: integrating cross-sectional and capture–recapture approaches to infer disease dynamics' Ecology 101: e02923', not Gamble et al. 2020 in 'J Appl Ecol'.

Author response: *We thank the reviewer for pointing out this oversight, we have corrected the citation to Gamble* et al. *2020 in* Ecology *101: e02923 in the revised manuscript.*

Reviewer #3 (Remarks to the Author):

I appreciate the authors addressing my concerns in their response to reviews.

Author response: *We thank the reviewer for their time and positive assessment of our revised manuscript.*
